# Diversity of Guilds of Amphibian Larvae in North-Western Africa

**DOI:** 10.1371/journal.pone.0170763

**Published:** 2017-01-26

**Authors:** Daniel Escoriza, Jihène Ben Hassine

**Affiliations:** 1 GRECO, Institute of Aquatic Ecology, University of Girona, Campus Montilivi, Girona, Spain; 2 Faculty of Sciences of Tunis, University Tunis El Manar, Tunis, Tunisia; 3 Laboratory Ecology, Biodiversity and Environment, Faculty of Sciences of Tétouan, University Abdelmalek Essaâdi, El M’Hannech II, Tetouan, Morocco; Universitat Zurich, SWITZERLAND

## Abstract

The composition and diversity of biotic assemblages is regulated by a complex interplay of environmental features. We investigated the influence of climate and the aquatic habitat conditions on the larval traits and the structure of amphibian larval guilds in north-western Africa. We classified the species into morphological groups, based on external traits: body shape, size, and the relative positions of the eyes and oral apparatus. We characterized the guild diversity based on species richness and interspecific phylogenetic/functional relationships. The larvae of the urodeles were classified as typical of either the stream or pond type, and the anurans as typical of either the lentic-benthic or lentic-nektonic type. The variations in the body shapes of both urodeles and anurans were associated with the type of aquatic habitat (lentic vs lotic) and the types of predators present. Most of the urodele guilds (98.9%) contained a single species, whereas the anuran guilds were usually more diverse. Both the phylogenetic and functional diversity of the anuran guilds were positively influenced by the size of the aquatic habitat and negatively by aridity. In anurans, the benthic and nektonic morphological types frequently co-occurred, possibly influenced by their opportunistic breeding strategies.

## Introduction

The factors regulating the variation of biotic communities along ecological gradients have been much studied in ecology because of their implications in understanding the processes underlying species associations [1,2]. Community diversity is determined by a complex interplay of habitat features, species interactions and present/historical climatic conditions [3,4]. This means that the variation in community diversity is not always simply correlated with environmental predictors, particularly over relatively large spatial scales [5].

We investigated the factors that might determine the composition and diversity of amphibian larval guilds in north-west Africa. Diversity can be described as species richness, but it is also related to the degree of functional or phylogenetic relatedness of the species forming a community, which can differ even in situations where the richness of two communities is almost equal [6]. Moreover assessing the phylogenetic and functional diversity of communities allows determine some mechanisms of the functionality of an ecosystem that do not depend solely on the number of species [7]. In anurans, species-rich communities are organized by the divergence of morphological traits, facilitating the occurrence of species at distinct habitat levels (like the heights of arboreal strata [8]), but there are also communities in which co-occurring species show high functional redundancy [9].

We examined the effects of climate and habitat type, because both are correlated with species composition in a community [10,11]. Furthermore, the region under study shows contrasting environmental clines associated with rapid spatial species turnover [12]. Therefore, we expected that the amphibian larval guilds would be significantly affected by the gradients in environmental factors, becoming more homogeneous (i.e., less functionally and phylogenetically diverse) under arid climates. Similarly, we expected that irrespective of climate, the types of aquatic habitats would also exert an important influence on community structure. Large water bodies, with high mesohabitat heterogeneity, host more diverse amphibian communities, up to a limit imposed by the increasing effects of predators and competition [13,14].

Amphibian larvae display a broad variation in shape, depending on whether they occur in lentic or lotic habitats, and where they live in the water column [15,16]. Therefore those species that occupy similar larval habitats also share some morphological traits, independent of their evolutionary relationships [17,18]. This allows species to be classified into morphological groups, which are similar in the distinct global ecoregions [17]. In temperate regions most urodeles can be grouped into two main groups [16]; in ‘stream’ group, the overall shape has a relatively shorter vertical axis and the dorsal fin is restricted to the tail, whereas in the ‘pond’ group, the overall shape has a relatively longer vertical axis and the dorsal fin extends along the back. Similarly, in anurans, in the ‘lentic-benthic’ group the overall shape has a relatively shorter vertical axis and in the ‘lentic-nektonic’ group the overall shape has a relatively longer vertical axis [17]. In north-western Africa, the genus *Salamandra* and *Pleurodeles* were assigned to stream and pond types respectively [19], but the morphological affinities of larval anurans have not yet been ascertained.

In this study, we tested several hypotheses regarding the variation in composition of larval amphibian communities. We expected that the different species occurring in north-western Africa would cluster into two main morphological groups, as in other temperate regions, and that group occurrence would be structured along an aquatic habitat gradient [20,21]. We expected that some larval traits (such as larval size, body shape, and developmental period) would correlate with the environmental cline; e.g., in the arid belt, the predominant anuran species would have benthic larvae that developed rapidly, traits that are usually associated with ephemeral ponds [22]. We expected that nektonic types, which occur in deeper water bodies [17], would be associated with more mesic conditions. We also expected clinal variations in the shapes of the urodele larvae, because the stream type of larvae is frequently associated with cooler and humid montane habitats, whereas the pond type is more typical of the plains [16]. Finally we predicted that this gradient would influence the larval assemblage composition, both in species identity and functional/phylogenetic diversity, becoming simpler under more extreme conditions.

## Methods

### Study Region

**Fig 1:**
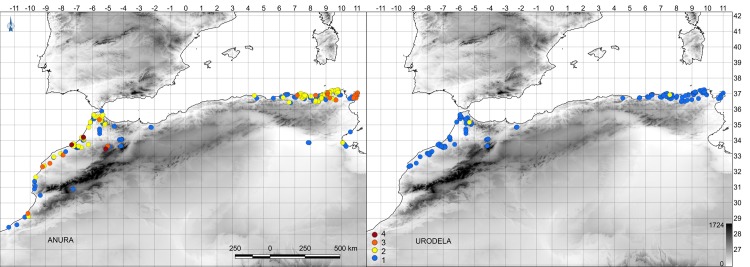
Map of the study region, showing sampled sites and amphibian species-richness at the larval stage.

Our study covered most of north-west Africa, including Morocco, Algeria and Tunisia (Fig 1). The region shows a marked variation in climate, from the humid regions near the Mediterranean coast (*Csa* climate type, Köppen classification) to the subtropical Sahara Desert (*BWh* climate type, Köppen classification) [23]. Hydrologically, the region is characterized by high seasonality and irregular precipitation, with occasional prolonged droughts [24]. Fourteen species of amphibians (four urodeles and ten anurans) are currently recognised in the region [25,26]. The eastern Algerian and Tunisian populations of *Hyla meridionalis* are genetically very different from the Moroccan populations, so we treated both clades separately [27]. Fieldwork was authorised by scientific permits provided by the Algerian authorities (University Badji Mokhtar, Annaba), the Tunisian Ministry of Agriculture and the Haut Commissariat aux Eaux et Forêts et à la Lutte Contre la Désertification (HCEFLCD/DLCDPN /CFF), Morocco. The field studies did not involve endangered or protected species.

### Sampling and Habitat Characterization

The aquatic habitats surveyed included temporary ponds, daïats (permanent/semi-permanent ponds), springs and streams. We defined ‘ponds’ as both artificial and natural temporary/permanent lentic water bodies, including gravel pits and cattle pools [28]; ‘springs’ as rheocrene/helocrene water sources that contain standing water or with low flow [29]; ‘streams’ as natural lotic water bodies, less than 8.25 m in width [28], and ‘stream pools’ as sections of a stream bounded by stone/ground margins or separated by different ground levels (preventing the exchange of amphibian larvae). These habitats were sampled in February-April over a 5–year period (2010–2015), based on previous surveys that showed that this period included the breeding activity of all the amphibians occurring in the region, although some species (such as *Amietophrynus mauritanicus*, *Discoglossus pictus*, and *Pelophylax saharicus*) can extend their reproductive period until the beginning of summer [30,31]. Given the way we collected data, we could not estimate detection probabilities. We therefore have to assume that detection probabilities were constant.

The presence of larvae was determined by dip netting. Dip net effort was set proportional to pond area using up to 10 dips in water bodies with surface areas < 50 m^2^, and up to 60 dips in water bodies > 1000 m^2^ (S1A Table). Sampling was performed across all heterogeneous mesohabitats, from the open to vegetated zones of the water body. In the case of the streams, the stones were turned over to increase the chances of capture. We also determined the occurrence of predators in the aquatic habitats. We classified them into four categories: native predatory arthropods (Dytiscidae, Heteroptera, Hydrophilidae, Libellulidae, Notostraca, *Potamon algeriense* [32]), alien species (*Gambusia holbrooki*, *Lepomis gibbosus*, *Procambarus clarkii*; [33,34]), urodeles (both larvae and adults), and semi-aquatic reptiles *(Emys orbicularis*, *Mauremys leprosa*, *Natrix astreptophora*, *Natrix maura;* [31]). All these species or taxonomic groups prey amphibian larvae on a regular basis and influence the compositions of amphibian communities [31,35–37]. The variables measured included the average water body surface area (m^2^) and water column depth (cm). The surface area of each water body was estimated by measuring the maximum length of its longitudinal axis and the length of its transverse axis, and assuming an elliptical shape. In water bodies larger than 100 m^2^, the surface area was estimated using a Garmin Dakota 100 GPS unit. Average depth was taken as the mean value of five successive measurements from the shore to the centre of the water body. We visually estimated the percentage of the surface area covered by emergent vegetation. We also examined water temperature (°C) *in situ* with a Hach HQ10 Portable LDO meter. The aquatic habitats were sampled between 11:00 h and 16:00 h (local time) in order to standardise the measurement.

### Geographic Information Systems Data

We used data taken from a set of variables that describe environmental conditions, and are relevant to amphibian natural history [38]. Forest cover was described based on remote sensing imagery, with a resolution of 30 m pixel^–1^ [39]. Topography was described based on a digital elevation model, with a resolution of 250 m pixel^–1^[40]. Climate was described based on mean annual temperature and a surrogate for the water-energy balance–the aridity index (mean annual precipitation/mean annual potential evapotranspiration) both with a resolution of 1000 m pixel^–1^[41,42]. The aridity index ranges from 0–0.03 (hyper-arid), 0.03–0.2 (arid), 0.2–0.5 (semi-arid), 0.5–0.65 (dry sub-humid), to ≥ 0.65 (humid) [42]. Data was extracted from GIS layers using the package Quantum-GIS vs 2.18 [43].

### Morphological Data and Groups

We described larval phenotypes (shape and size) based on analyses of digital images. To reduce the variability associated with the developmental changes of the larvae, we included only those larvae in the advanced stages of development: Gallien-Durocher stages 55a and 55b in *Pleurodeles* and Gosner stages 30–38 in anuran larvae [44,45]. For each species, we selected specimens from distinct types of aquatic habitat whenever possible, in an attempt to capture the range of phenotypic diversity. The site and aquatic habitat characteristics and the number of specimens per species examined are shown in S1A and S1B Table. Photographs were taken following a standardized protocol [46]. We used these images to perform an outline shape analysis based on discrete Fourier transformations [47]. Images from each specimen were decomposed into 80 Elliptic Fourier Coefficients, which allow the definition of species’ mean shapes [47]. The 80 coefficients characterizing the species’ mean shapes were then included in a hierarchical agglomerative clustering obtained by multiscale bootstrap resampling [48]. This method estimates the optimal number of clusters and their statistical likelihoods, based on approximately unbiased (AU) *P*-values [48]. The species’ clusters, generated separately for both taxonomic orders, were interpreted as the principal larval types or morphological groups. To assess the possible variation associated with phenotypic plasticity, we estimated the occurrence of any significant intraspecific variability with permutational multivariate analysis of variance (PERMANOVA) [49]. Image analyses were conducted using the package SHAPE vs. 1.3 [50] and ImageJ vs 1.50e [51]. Statistical analyses were carried out using the package ‘pvclust’ [52] for R [53] and PRIMER-E (PRIMER-E Ltd., Plymouth).

We included larval traits other than body shape and size in the analyses: the larval development time, the relative positions of the eyes and the oral apparatus, and the colour pattern on the tail [17]. The time required to complete larval development is a critical factor determining the occurrence of amphibians in aquatic habitats, and different species vary greatly in these periods, according to their different reproductive strategies [17]. The positions of the eyes (dorsal or lateral) and oral apparatus (anteroventral or terminal) are traits linked to body shape, and vary according to the species’ occupancy of the water column [17]. The tail colour pattern (bicolored, mottled, or uniform) can indicate differences in the larval exposure to predators [54]. These data were obtained from bibliographic sources [31,55] and are shown in S1B Table. In the urodeles studied, there were no differences in the relative positions of the eyes or mouth (S1B Table), so these traits were not considered in the analysis.

### Guild Structure

We defined ‘guild’ as a group of related species that co-occur in a single aquatic habitat and therefore share similar resources, without defining the functional roles of the species composing that community [56]. Because both taxonomic orders (urodeles and anurans) occupy different trophic levels, we studied them separately. Urodele larvae are carnivorous, and their diets include several types of arthropods and anuran larvae, whereas most anuran larvae are omnivorous [18].We assessed the guild diversity based on the species richness, phylogenetic distances and functional indices.

The degree of phylogenetic relatedness among the species comprising a guild was estimated using the mean pairwise phylogenetic distance [57]. The phylogenetic distances among taxa (measured in millions of years ago; Ma) were obtained from TimeTree, based on the median distances of the molecular time estimates [58]. The relative functional distances based on the scores of the first axis of Principal Component Analyses (PCA) of the species’ mean shapes, the species sizes, the development time and the categorical traits were obtained by Gower’s similarity coefficient [59]. We calculated two measures of functional evenness–the species average distance to the group centroid, and the nearest neighbour distance in the principal coordinates (PCO) plane [59]. To assess guild diversity, we selected those indices that measure functional evenness, because they are less biased by species richness [60]. All these calculations were performed using the package PRIMER-E (PRIMER-E Ltd., Plymouth).

### Data Analysis

We investigated three possible effects of gradient on the composition of larval guilds: (i) on species identity; (ii) on larval traits; and (iii) on diversity. First, we examined the environment influence on species identity using a Canonical Outlying Mean Index analysis (CANOMI) [61]. This type of analysis evaluates the importance of the variables explaining species occurrence, but also the deviation (or marginality) of these species relative to a hypothetical average niche [61]. We were also interested in visualizing groups of species using average clustering, based on CANOMI scores, identifying significant groups based on similarity profile permutation test [62]. Differences in the compositions of larval guilds within the same type of aquatic habitat were evaluated with PERMANOVA, using similarity matrices computed with Sørensen distances. In this analysis, we took into account the variability attributable to environmental conditions. To do so, we constructed a model that included the elevation, mean annual temperature, aridity index, and forest cover as covariates.

We examined the possible correlation between larval traits and the environmental gradient using the ‘fourth-corner’ approach [63]. This method assesses the relationship between three matrices: (i) a presence-absence matrix; (ii) an environmental matrix, containing the predictor data; and (iii) a trait matrix. In this analysis, we also included the categories of predators because they have different impact on larval types [64]. We were also interested in assessing the phylogenetic structure of the larval morphospace. To do this, we assessed the associations between phylogenetic distances and continuous traits (body shape and size) using Moran’s autocorrelation coefficient (Moran’s *I*) [65]. This coefficient ranges from −1 (the species are less similar than expected under a Brownian motion model) and 1 (the species are more similar than expected) [65].

We modeled the association between the gradient and diversity (species richness, taxonomic and functional indices) using Generalized Linear Models (GLMs). We constructed the GLMs using Poisson (species richness) and Gaussian distributions (diversity indices). The descriptors of aquatic habitats (surface area and average depth) were normalized and transformed to a single variable using a PCA. The best explanatory models were selected based on Akaike’s Information Criterion with a correction for finite sample sizes (AICc) [66]. The models were compared using AICc values and two associated measures: delta AICc and Akaike weights. In general, a delta value < 2 is strong evidence for the model [66]. Akaike weights are a measure of the relative importance of a variable, and are the sum of the variable weights in all the models containing that variable [66]. These analyses were conducted using the ‘ade4’ [67], ‘adehabitatHS’ [68] and ‘MuMIn’ [69] packages in R.

## Results

We detected the presence of amphibian larvae in a large part of the study area (Fig 1). In most of the water bodies surveyed urodele guilds included only a single species (98.9%), whereas in anurans 53.6% of water bodies comprised one species, 38.9% two species, 6.1% three species, and 1.4% four species. The characteristics of the water bodies and sites where each species occurred (Tables 1 and 2) showed that some species tolerate a wide variety of environmental parameters, particularly some bufonids and ranids such *Amietophrynus mauritanicus*, *Bufotes boulengeri* and *Pelophylax saharicus*.

**Table 1:** Water body characteristics (mean and standard deviation) for north-western Africa amphibians. Surface area (m^2^); average depth (cm); water temperature (°C).

	Surface area	Average depth	Emergent vegetation	Water temperature
*Alytes maurus*	32 ± 13	23 ± 6	0.5 ± 0.29	12.8 ± 1.6
*Amietophrynus mauritanicus*	3586 ± 1496	111 ± 78	0.4 ± 0.06	20.6 ± 0.8
*Barbarophryne brongersmai*	9240	9	0.2	18.9
*Bufo spinosus*	2038 ± 869	38 ± 16	0.4 ± 0.18	12.5 ± 2.4
*Bufotes boulengeri*	4.6e04 ± 4.3e04	27 ± 4	0.3 ± 0.06	21.8 ± 0.6
*Discoglossus pictus*	1304 ± 283	29 ± 2	0.6 ± 0.03	17.7 ± 0.4
*Discoglossus scovazzi*	6059 ± 4144	28 ± 4	0.5 ± 0.06	14.7 ± 0.8
*Hyla aff*. *meridionalis*	917 ± 177	80 ± 40	0.5 ± 0.04	17.2 ± 0.5
*Hyla meridionalis*	2.2e04 ± 1.2e04	34 ± 2	0.7 ± 0.03	16.8 ± 0.4
*Pelobates varaldii*	4679 ± 1712	38 ± 3	0.7 ± 0.06	16.9 ± 0.5
*Pelophylax saharicus*	2.6e04 ± 2.3e04	94 ± 53	0.5 ± 0.05	17.7 ± 0.7
*Pleurodeles nebulosus*	808 ± 232	27 ± 3	0.5 ± 0.04	19,2 ± 0.6
*Pleurodeles poireti*	3037 ± 1049	37 ± 5	0.7 ± 0.06	13.9 ± 0.5
*Pleurodeles waltl*	3.2e04 ± 1.3e04	31 ± 2	0.7 ± 0.03	17.4 ± 0.4
*Salamandra algira*	357 ± 120	32 ± 4	0.3 ± 0.06	11.3 ± 0.4

**Table 2:** Niche characteristics (mean and standard deviation) for north-western Africa amphibians. Elevation (m); annual temperature (°C); forest cover (% per 30 m).

	Elevation	Annual temperature	Aridity index	Forest cover
*Alytes maurus*	1550 ± 162	11.6 ± 1.0	0.65 ± 0.14	37 ± 18
*Amietophrynus mauritanicus*	176 ± 46	17.8 ± 0.1	0.36 ± 0.03	0 ± 0
*Barbarophryne brongersmai*	187	17.3	0.27	0
*Bufo spinosus*	965 ± 227	14.0 ± 0.9	0.84 ± 0.04	36 ± 15
*Bufotes boulengeri*	167 ± 58	18.0 ± 0.3	0.31 ± 0.03	0 ± 0
*Discoglossus pictus*	153 ± 19	17.6 ± 0.1	0.56 ± 0.02	8 ± 2
*Discoglossus scovazzi*	377 ± 100	16.8 ± 0.4	0.61 ± 0.41	12 ± 5
*Hyla aff*. *meridionalis*	145 ± 28	17.7 ± 0.1	0.58 ± 0.02	6 ± 3
*Hyla meridionalis*	224 ± 36	17.5 ± 0.1	0.45 ± 0.02	4 ± 1
*Pelobates varaldii*	96 ± 9	18.2 ± 0.0	0.46 ± 0.05	0 ± 0
*Pelophylax saharicus*	371 ± 71	16.9 ± 0.3	0.50 ± 0.03	17 ± 5
*Pleurodeles nebulosus*	108 ± 23	17.7 ± 0.1	0.53 ± 0.03	5 ± 3
*Pleurodeles poireti*	55 ± 14	17.1 ± 0.1	0.64 ± 0.01	2 ± 1
*Pleurodeles waltl*	159 ± 14	17.9 ± 0.1	0.41 ± 0.01	1 ± 1
*Salamandra algira*	800 ± 67	14.9 ± 2.9	0.71 ± 0.04	42 ± 6

**Fig 2:**
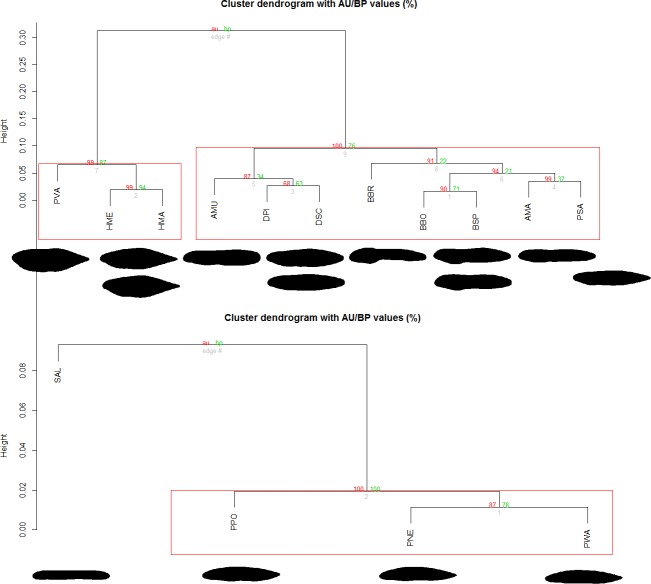
Hierarchical clustering based on multiscale bootstrap resampling of Elliptic Fourier Coefficients. AU (Approximately Unbiased) *P*-value and BP (Bootstrap Probability) value. The main groups can be assigned to different morphological groups, marked with a red square. AMA, *A*. *mauritanicus*; AMU, *A*. *maurus*; BBO, *B*. *boulengeri*; BBR, *B*. *brongersmai*; BSP, *B*. *spinosus*; DPI, *D*. *pictus*; DSC, *D*. *scovazzi*; HMA, *H*. *aff*. *meridionalis* (eastern form); HME, *H*. *meridionalis*; PNE, *P*. *nebulosus*; PPO, *P*. *poireti*; PSA, *P*. *saharicus*; PVA, *P*. *varaldii*; PWA, *P*. *waltl*; SAL, *S*. *algira*.

The hierarchical agglomerative analysis of the species mean shapes showed two highly supported clusters in urodeles (AU *P*-values = 1.00), separating *Salamandra* from the *Pleurodeles* species (Fig 2). *Pleurodeles* larvae were characterized by their overall shapes having a relatively longer vertical axis, and large dorsal fins (Fig 2). *Salamandra algira* showed a shorter vertical axis and a short caudal fin. In anurans, we detected two supported clusters (AU *P*-values = 1.00), separating the genus *Hyla* and *Pelobates* from *Alytes*, *Amietophrynus*, *Barbarophryne*, *Bufo*, *Bufotes*, *Discoglossus*, and *Pelophylax* (Fig 2). *Hyla meridionalis* (both sublineages) and *Pelobates varaldii* larvae were characterized by their overall shapes having a relatively longer vertical axis and large dorsal fins (Fig 2). The remaining species showed shorter vertical axis and short caudal fins (Fig 2). PERMANOVA showed no significant intraspecific variability (S1C Table). There was also no correlation between the morphological traits and phylogenetic distances in the urodeles (body shape, Moran’s *I* = –0.825, *P* = 1.000; size, Moran’s *I* = –0.488, *P* = 0.504) and anurans (body shape, Moran’s *I* = –0.145, *P* = 0.746; size, Moran’s *I* = –0.210, *P* = 0.927).

**Fig 3:**
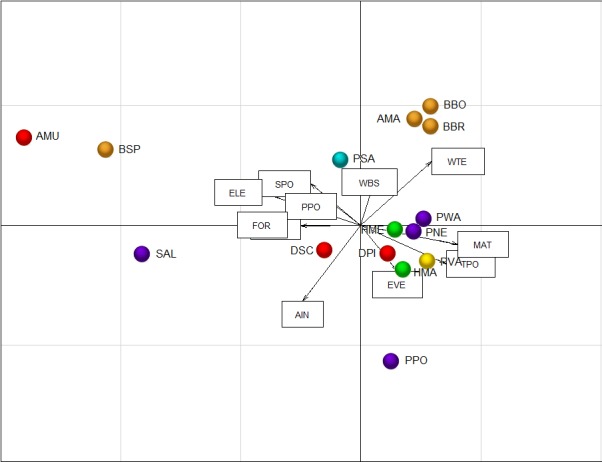
Canonical Outlying Mean Index ordination plot of sites of species occurrence, with the environmental variables fitted as vectors. WBS, water body size; WTE, water temperature; EVE, emergent vegetation; ELE, elevation; MAT, mean annual temperature; AIN, aridity index; FOR, forest cover; PPO, permanent ponds; SPO, stream pools; SPR, springs; TPO, temporary ponds. AMA, *A*. *mauritanicus*; AMU, *A*. *maurus*; BBO, *B*. *boulengeri*; BBR, *B*. *brongersmai*; BSP, *B*. *spinosus*; DPI, *D*. *pictus*; DSC, *D*. *scovazzi*; HMA, *H*. *aff*. *meridionalis* (eastern form); HME, *H*. *meridionalis*; PNE, *P*. *nebulosus*; PPO, *P*. *poireti*; PSA, *P*. *saharicus*; PVA, *P*. *varaldii*; PWA, *P*. *waltl*; SAL, *S*. *algira*.

**Fig 4:**
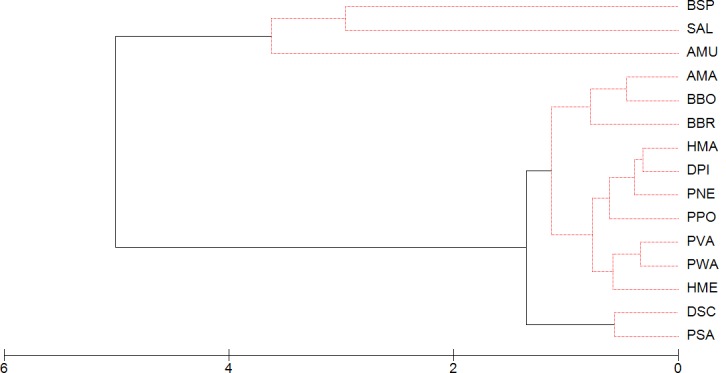
Main ecological groups of north-western African amphibians obtained by group average clustering of the species OMI coordinates. π statistics obtained by the SIMPROOF test showed significant clusters at *P* ≤ 0.05 (bold line). AMA, *A*. *mauritanicus*; AMU, *A*. *maurus*; BBO, *B*. *boulengeri*; BBR, *B*. *brongersmai*; BSP, *B*. *spinosus*; DPI, *D*. *pictus*; DSC, *D*. *scovazzi*; HMA, *H*. *aff*. *meridionalis* (eastern form); HME, *H*. *meridionalis*; PNE, *P*. *nebulosus*; PPO, *P*. *poireti*; PSA, *P*. *saharicus*; PVA, *P*. *varaldii*; PWA, *P*. *waltl*; SAL, *S*. *algira*.

The first axis of the CANOMI (CS1; eigenvalue = 0.43) showed a highly negative correlation to the aridity index, elevation, forest cover and some aquatic habitat types (springs and stream pools) and positive to mean annual temperature and temporary ponds (Fig 3 and Table 3). This axis described the transition from mountains to plains. The species that showed a high negative correlation with CS1 were *Salamandra algira*, *Alytes maurus* and *Bufo spinosus* (Fig 3). These species occupied marginal positions in the niche space, relegated to humid mountain forests (S1D Table), and comprised a distinctive group from other North African amphibians (Figs 3 and 4). The second axis of the CANOMI (CS2; eigenvalue = 0.19) was negatively associated with the aridity index and positively to water temperature (Fig 3 and Table 3). This axis described the transition from humid to arid conditions. Species occurring in arid conditions (*Amietophrynus mauritanicus*, *Barbarophyrne brongersmai*, *Bufotes boulengeri* and *Pelophylax saharicus*) showed a highly positive correlation with CS2, whereas the genera *Pleurodeles*, *Hyla*, *Discoglossus* and *Pelobates* showed a negative relationship (Fig 3). PERMANOVA showed that the relationships between the assemblage composition and the mean temperature and the type of aquatic habitat were statistically significant, although they were determined by the aridity gradient within the same type of aquatic habitat (Table 4).

**Table 3:** Results of the canonical outlying mean index assessing the influence of the environmental gradient in the species occurrence. Eigenvalues and factor scores for the environmental variables are provided for the first two axes (CS1 and CS2).

		CS1	CS2
Eigenvalues		0.43	0.19
Water body size		0.07	0.25
Water temperature		0.59	0.53
Emergent vegetation		0.31	–0.39
Elevation		–0.83	0.27
Mean annual temperature		0.81	–0.16
Aridity index		–0.49	–0.64
Forest		–0.62	–0.001
Habitat type	Permanent ponds	–0.20	0.16
	Stream pools	–0.42	0.35
	Springs	–0.50	–0.01
	Temporary ponds	0.71	–0.32

**Table 4:** PERMANOVA test assessing differences in the composition of amphibian guilds depending on the types of aquatic habitats and macro-environmental descriptors, including the interactions between parameters. Probabilities are shown in bold when *P* ≤ 0.05.

Parameters	Pseudo-*F*	*P*
Water body type	1.80	**0.0400**
Elevation	0.71	0.6011
Elevation x water body type	0.48	0.9343
Mean annual temperature	2.43	**0.0443**
Mean annual temperature x water body type	0.97	0.4665
Aridity index	0.62	0.6722
Aridity index x water body type	2.14	**0.0097**
Forest cover	2.14	0.0655
Forest cover x water body type	0.93	0.5199

A fourth-corner analysis showed that in urodeles, body shape was influenced by the aquatic habitat type, water temperature and the predator type, whereas in anurans, it was influenced by the aquatic habitat type, emergent vegetation and the predator type (Tables 5 and 6). A marginally significant correlation was detected between the body shape of the anuran larvae and the aridity index and forest cover (Table 6). As expected, the relative positions of the eyes and oral apparatus showed correlations similar to those of body shape in the anuran larvae, and were associated with emergent vegetation and predator type (Table 6). The best GLM explaining the variation in species richness included the water body size (positive relationship; Table 7). The best model explaining the variation in mean phylogenetic distances included the aridity index, water body size, water temperature, temporary ponds (positive relationship) and forest cover (negative relationship). The best model explaining the variation in functional indices included the water body size, aridity index, temporary ponds, and emergent vegetation (the latter only explained the nearest neighbour functional distance; Table 7) which showed positive relationships.

**Table 5:** Correlations of urodele larvae traits with the environmental gradient, based on fourth-corner analysis. Water body size, PC1 scores of water mass surface area and average depth. Body shape, PC1 scores of mean shapes, obtained by Fourier transformation. Probabilities are shown in bold when *P* ≤ 0.05.

		Body shape	Larval size	Development time	Color pattern
Water body size	correlation	–0.09	–0.05	–0.15	1.17
	*P*	0.253	0.520	0.334	0.286
Water temperature	correlation	–0.61	0.13	–0.64	95.02
	*P*	**0.041**	0.086	0.334	0.250
Emergent vegetation	correlation	–0.38	0.27	–0.28	33.10
	*P*	0.168	0.374	0.504	0.250
Predator type	correlation	46.42	15.63	18.53	62.94
	*P*	**0.041**	0.417	0.334	0.250
Habitat type	correlation	–0.61	26.99	31.18	96.23
	*P*	**0.041**	0.374	0.334	0.250
Elevation	correlation	0.77	–0.56	0.49	278.18
	*P*	0.166	0.292	0.334	0.250
Mean temperature	correlation	–0.77	0.56	–0.50	273.30
	*P*	0.250	0.292	0.334	0.250
Aridity index	correlation	0.48	–0.09	0.547	50.79
	*P*	0.379	0.248	0.334	0.505
Forest cover	correlation	0.65	–0.44	0.46	136.79
	*P*	0.168	0.374	0.334	0.250

**Table 6:** Correlations of anuran larvae traits with the environmental gradient, based on fourth-corner analysis. Water body size, PC1 scores of water mass surface area and average depth. Body shape, PC1 scores of mean shapes, obtained by Fourier transformation. Probabilities are shown in bold when *P* ≤ 0.05.

		Body shape	Larval size	Development time	Eye/Oral apparatus	Color pattern
Water body size	Correlation	0.01	0.08	0.12	2.05	3.76
	*P*	0.852	0.070	**0.033**	0.120	0.085
Water temperature	correlation	–0.15	–0.04	–0.09	4.62	2.67
	*P*	0.146	0.344	0.052	0.381	0.669
Emergent vegetation	correlation	0.31	0.11	0.16	3.15	1.93
	*P*	**0.004**	0.344	0.253	**0.024**	0.085
Predator type	correlation	11.44	1.31	2.34	35.21	8.49
	*P*	**0.0004**	0.532	0.329	**0.004**	0.813
Habitat type	correlation	9.62	0.36	1.89	17.04	8.60
	*P*	**0.024**	0.776	0.094	0.149	0.082
Elevation	correlation	–0.16	0.00	0.02	4.73	1.29
	*P*	0.114	0.939	0.595	0.352	0.186
Mean temperature	correlation	–0.15	0.02	–0.00	5.26	1.11
	*P*	0.146	0.584	0.936	0.311	0.240
Aridity index	correlation	0.09	–0.05	–0.01	0.39	11.71
	*P*	0.051	0.257	0.841	0.488	0.325
Forest cover	correlation	–0.09	0.00	0.02	6.91	1.64
	*P*	0.053	0.937	0.568	0.119	0.123

**Table 7:** Relative importance (weighted) of the variables included in the best supported GLMs (Delta AICc < 2) testing for the patterns of phylogenetic and functional structure of anuran guilds. Variables with weights greater than 0.5 are shown.

Diversity indices	Parameters	Coefficients	Weight
Species richness	Water body size	0.074	0.91
Mean phylogenetic distance	Aridity index	0.197	1.00
	Water body size	0.114	0.91
	Water temperature	0.132	0.68
	Forest cover	–0.121	0.57
	Temporary pond	0.126	0.52
Mean functional distance	Water body size	0.205	1.00
	Temporary pond	0.135	0.98
	Aridity index	0.106	0.73
Nearest functional distance	Water body size	0.174	1.00
	Aridity index	0.132	0.95
	Temporary pond	0.136	0.89
	Emergent vegetation	0.080	0.52

## Discussion

Compared with other Mediterranean regions, only a small number of amphibian species inhabit north-west Africa [70,71]. Here we present the first comprehensive assessment of the factors determining the phylogenetic and functional diversity of the amphibian guilds in north-western Africa.

The analyses showed two well-supported morphological clusters in urodeles. The larvae of one urodele group (which included only one species, *Salamandra algira*) were characterized by a flattened shape, uniform coloration, and relatively small size. These characters, together with others (dorsal fin ending at the base of the tail, gills reduced, and the presence of functional limbs at hatching), indicate that this species can be assigned to the stream type [19]. The larvae of the other group, which included the three species of the genus *Pleurodeles*, were characterized by a fusiform shape, speckled tail pattern, and larger size. These characters, together with the presence of long gills, a dorsal fin that extended to the back, and the absence of functional limbs at hatching, suggest that these species be included within the pond morphological type [19].

The stream type also occupies lentic habitats in north-western Africa. This has also been reported for other salamanders grouped within the stream type (e.g., in *Desmognathus*, *Euproctus*, *Eurycea*, *Necturus* and *Salamandra* species; [16,72,73]). By contrast, the pond type rarely occupies lotic habitats in north-western Africa. The partitioning of the use of lotic and lentic habitats, together with differences in the optimal temperatures for larval development [30], possibly explains the little overlap observed between the two urodele morphological groups in the region (1.1% of aquatic habitats). Predators could also have some influence on these patterns because *Pleurodeles* larvae frequently occur in habitats where there is abundance of large arthropods. The high dorsal fins of the *Pleurodeles* larvae probably make them less vulnerable to attack by this type of predators [74]. The low spatial overlap observed between *Pleurodeles nebulosus* and *Pleurodeles poireti* was probably caused by competitive interference [75].

In anura we also detected in two well-supported morphological clusters. One anuran larval group was characterized by a fusiform shape, with a high vertical/horizontal ratio, lateral eyes, an oral apparatus situated in a terminal position, moderate-large size, and a long period of larval development. This group included the genera *Hyla* and *Pelobates*, and corresponded to the lentic-nektonic type [17]. The other group was characterized by its flattened form, dorsal eyes, and oral apparatus located in the antero-ventral region. This group included the genera *Alytes*, *Amietophrynus*, *Barbarophryne*, *Bufo*, *Bufotes*, *Discoglossus*, and *Pelophylax*, and corresponded to the lentic-benthic type [17].

The occurrence of both main morphological types was influenced by the aridity and the level of forest cover in the landscape. Increased aridity is associated with unstable hydrological regimes, which possibly favour species with small benthic tadpoles, such as xeric Bufonidae (genera *Barbarophryne* and *Bufotes*) and *Discoglossus* [31]. At the other end of the gradient, the effect of the forest cover may be attributable to the fact that only a few species in the region can complete their development in shaded ponds (*Alytes maurus* and *Bufo spinosus*, lentic-benthic group). Some features of the aquatic habitats also affected the occurrence of both anuran morphological groups. The lentic-nektonic group (genera *Hyla* and *Pelobates*) showed a positive association with temporary ponds densely populated by emergent macrophytes (*Isoetes*, *Ranunculus*). These aquatic habitats are particularly favourable for nektonic species because they are fishless, have relatively long hydroperiods (necessary for the development of macrophyte communities [76]), and provide shelter and food for large tadpoles [77]. In these ponds nektonic species frequently coexist with the lentic-benthic group (*Hyla* and *Discoglossus* species appear together in 21% of the surveyed ponds). However, the lentic-benthic group was not associated with specific habitat features, and some species in this group are generalists that breed in a broad range of aquatic habitats (e.g., *Discoglossus pictus* and *Pelophylax saharicus*).

Larval size is not related to differences in habitat occupancy. Greater size in anuran larvae represents an adaptive advantage in those aquatic habitats hosting a high density of predators, which are more common in larger water bodies [78,79]. This reproductive strategy also has its cost, because larger tadpoles require longer hydroperiods to complete their development [80]. Similarly the two species in the region with very large larvae are associated with permanent or long-hydroperiod ponds [31,81]. However, although *Pelophylax* and *Pelobates* larvae may be better adapted to occupy these habitat types, other species are not completely excluded, even those that typically breed in ephemeral pools. In this sense, some species with short developmental periods (i.e., 15–60 days), such as *Discoglossus* and xeric Bufonidae [31,82], also occupy large aquatic habitats.

We detected no phylogenetic signal within the larval morphospace. Distantly related species display similar phenotypes (e.g., comparing *Discoglossus* and Bufonidae, separated by 211 Ma [58]). Unstable climatic behaviour over long periods [83,84] could have favoured those species with generalist phenotypes, as is observed in amphibians in other ecozones [85,86].

The species composition of the larval guilds was largely influenced by the type of aquatic habitat and the climate, as expected. The aridity determined the species turnover in aquatic habitats (e.g., *Pelophylax saharicus* replaces *Alytes maurus* in streams along an aridity cline). The cluster-analyses indicated two main ecological groups in the region. One comprises *Alytes maurus*, *Bufo spinosus* and *Salamandra algira*, occupying marginal niches under cool, humid climates. These species belong to lineages of Eurasian origin, which invaded the region during the Late Neogene [87–89], and occupy similar habitats in northern Africa to those inhabited by congeneric species on the Iberian Peninsula. Both *Alytes maurus* and *Bufo spinosus* typically breed in low-order streams, although they lack the anatomical traits of true rheophilic tadpoles [17]. The other cluster is composed of eurytopic/thermophilic species, differentiated by their tolerance to aridity. Most of these species appear on steppes or in highly anthropic landscapes with no or low forest cover. This group frequently occupies lentic habitats, mainly temporary ponds, although *Pelophylax saharicus* also occurs in lotic habitats. *Amietophrynus mauritanicus*, *Barbarophryne brongersmai*, *Bufotes boulengeri*, *Discoglossus pictus*, *Hyla meridionalis* and *Pelophylax saharicus* favoring the presence of river networks reach extreme habitats in the margins of the Sahara Desert [26,31].

Temporary ponds covered with dense layers of macrophytes host the most complex tadpole guilds in the region. These results could be related to the absence of fish, greater primary productivity, greater structural complexity and the temporal variability of these aquatic habitats [28,90]. The size of the water bodies exerts little influence on the species found, but is the best predictor of the diversity of the larval guilds. This suggests that in north-western Africa amphibians try to maximize their use of the available water bodies. This may be due to the unpredictable precipitation in the region [91]. Most of these species breed synchronously, their reproductive behavior being triggered by the onset of the rains [31]. Opportunistic breeding is the most adaptive response to erratic rainfall patterns, and is widespread in those amphibians exposed to highly seasonal climates [92,93]. Low species richness (i.e., unsaturated guilds) could also favour stochastic associations between species [94]. Moreover, the abundance of predators in large aquatic habitats diminishes tadpole densities and the competition among tadpoles [95].

Higher species richness with increasing size of water body is also associated with higher phylogenetic and functional diversity of the tadpole guilds. Phylogenetic diversity was positively associated with the breadth of the functional space because numerous traits showed a phylogenetic signal [96]. These facts suggest that there may be some level of resource partitioning, possibly at the foraging level (i.e., benthic *vs*. pelagic foraging). This segregation at fine spatial scale between guilds is observed in other temperate amphibian larval communities [97] and possibly reduces interference between species, a critical aspect in ponds that may quickly dry out.

The species richness of amphibian larval guilds in north-western Africa is mainly determined by the size of the available aquatic habitats. Similar correlations have also been described in other ecoregions of the world, including highly diverse tropical communities [98,99]. This suggests that the size of aquatic habitats is a key factor structuring larval communities at the global scale, possibly facilitated by resource partitioning at several habitat levels [17]. In our study region, distinct anuran types tend to occur syntopically, possibly caused by opportunistic breeding under unpredictable rainfall regimes.

## Supporting Information

S1 Table Table A. Sites and species occurrence. Table B. Larvae traits. Table C. PERMANOVA results assessing intraspecific body shape variation. Table D. Canonical correlation between environmental variables and species occurrence.
(DOCX)

## References

[1] Rosenzweig ML, Sterner PW. Population ecology of desert rodent communities: body size and seed-husking as bases for heteromyid coexistence. Ecology. 1970; 1:217–224.

[2] Rahbek C. The elevational gradient of species richness: a uniform pattern?. Ecography. 1995; 18(2):200‒205.

[3] Kerr JT, Packer L. Habitat heterogeneity as a determinant of mammal species richness in high-energy regions. Nature. 1997; 385(6613):252‒254.

[4] Graham CH, Moritz C, Williams SE. Habitat history improves prediction of biodiversity in rainforest fauna. Proc. Nat. Acad. Sci. USA. 2006; 103(3): 632–636. 10.1073/pnas.0505754103 16407139 PMC1334636

[5] Gentry AH. Changes in plant community diversity and floristic composition on environmental and geographical gradients. Ann. Mo. Bot. Gard. 1988; 75:1–34.

[6] Laliberté E, Wells JA, DeClerck F, Metcalfe DJ, Catterall CP, Queiroz C, et al Land-use intensification reduces functional redundancy and response diversity in plant communities. Ecol. Lett. 2010; 13(1):76–86. 10.1111/j.1461-0248.2009.01403.x 19917052

[7] Hector A, Schmid B, Beierkuhnlein C, Caldeira MC, Diemer M, Dimitrakopoulos PG, et al Plant diversity and productivity experiments in European grasslands. Science. 1999; 286(5442):1123–1127. 10550043 10.1126/science.286.5442.1123

[8] Ernst R, Linsenmair KE, Rödel MO. Diversity erosion beyond the species level: dramatic loss of functional diversity after selective logging in two tropical amphibian communities. Biol. Conserv. 2006; 133(2):143–155.

[9] Strauß A, Reeve E, Randrianiaina RD, Vences M, Glos J. The world's richest tadpole communities show functional redundancy and low functional diversity: ecological data on Madagascar's stream-dwelling amphibian larvae. BMC Ecol. 2010; 10:12 10.1186/1472-6785-10-12 20459864 PMC2877654

[10] Davidson C, Bradley Shaffer H, Jennings MR. Declines of the California red-legged frog: climate, UV-B, habitat, and pesticides hypotheses. Ecol. Appl. 2001; 11(2):464–479.

[11] Hazell D, Cunnningham R, Lindenmayer D, Mackey B, Osborne W. Use of farm dams as frog habitat in an Australian agricultural landscape: factors affecting species richness and distribution. Biol. Conserv. 2001; 102:155–169.

[12] Escoriza D, Ruhí A. Macroecological patterns of amphibian communities in the Western Palearctic: Implications for conservation. Biol. Conserv. 2014; 176:252–261.

[13] Welborn GA, Skelly DK, Werner EE. Mechanisms creating community structure across a freshwater habitat gradient. Annu. Rev. Ecol. Syst. 1996; 27:337–363.

[14] Beja P, Alcazar R. Conservation of Mediterranean temporary ponds under agricultural intensification: an evaluation using amphibians. Biol. Conserv. 2003; 114:317‒326.

[15] Altig R, Johnston GF. Guilds of anuran larvae: relationships among developmental modes, morphologies, and habitats. Herpetol. Monog. 1989; 3:81‒109.

[16] Petranka JW. Salamanders of the United States and Canada. Washington: Smithsonian Institution Press; 1998.

[17] McDiarmid RW, Altig R. Tadpoles: the Biology of Anuran Larvae. Chicago: University of Chicago Press; 1999.

[18] Wells KD. The Ecology and Behavior of Amphibians. Chicago: University of Chicago Press; 2007.

[19] Ben Hassine J, Escoriza D. New ecological data on the family Salamandridae in the Maghreb. Herpetol. Rev. 2014; 45(2):193–200.

[20] Peltzer PM, Lajmanovich RC. Anuran tadpole communities in riparian areas of the Middle Parana River, Argentina. Biodivers. Conserv. 2004; 13(10):1833–1842

[21] Both C, Solé M, Dos Santos TG, Cechin SZ. The role of spatial and temporal descriptors for neotropical tadpole communities in southern Brazil. Hydrobiologia. 2009; 624:125–138.

[22] Newman RA. Adaptive plasticity in development of *Scaphiopus couchii* tadpoles in desert ponds. Evolution. 1988; 42(4):774‒783. 28563867 10.1111/j.1558-5646.1988.tb02495.x

[23] Peel MC, Finlayson BL, McMahon TA. Updated world map of the Köppen-Geiger climate classification. Hydrol. Earth Syst. Sci. Discuss. 2007; 4(2):439–473.

[24] El Garouani A, Tribak A. Relation entre hydrologie et climat dans le bassin versant de l’Oued Innaoune (pré-Rif Marocain). Int. Assoc. Hydrol. Sci. Publ. 2006; 308:447‒453.

[25] Bons J, Geniez P. Amphibians and Reptiles of Morocco (including Western Sahara), Biogeographical Atlas. Barcelona: Asociación Herpetológica Española; 1996.

[26] Ben Hassine J, Nouira S. Répartition géographique et affinités écologiques des Amphibiens de Tunisie. Rev. Écol. (Terre & Vie). 2012; 67:437–457.

[27] Recuero E, Iraola A, Rubio X, Machordom A, García-París M. Mitochondrial differentiation and biogeography of *Hyla meridionalis* (Anura: Hylidae): an unusual phylogeographical pattern. J. Biogeogr. 2007; 34(7):1207‒1219.

[28] Williams P, Whitfield M, Biggs J, Bray S, Fox G, Nicolet P, et al Comparative biodiversity of rivers, streams, ditches and ponds in an agricultural landscape in Southern England. Biol. Conserv. 2004; 115(2):329–341.

[29] Cantonati M, Gerecke R, Bertuzzi E. Springs of the Alps-sensitive ecosystems to environmental change: from biodiversity assessments to long-term studies. Hydrobiologia. 2006; 562(1):59–96.

[30] Escoriza D, Ben Hassine J. Niche partitioning at local and regional scale in the north African Salamandridae. J. Herpetol. 2015; 49:276–283.

[31] Schleich HH, Kastle W, Kabisch K. Amphibians and Reptiles of North Africa. Kögnistein: Koeltz Scientific; 1996.

[32] Tachet H, Richoux P, Bournaud M, Usseglio-Polatera P. Invertébrés d'Eau Douce: Systématique, Biologie, Écologie (Vol. 15). Paris: CNRS editions; 2010.

[33] Azeroual A. Monographie des poissons des eaux continentales du Maroc: systématique, distribution et écologie. M.Sc. Thesis, Université Mohammed V-Agdal. 2003. Available: http://abhatoo.net.ma/.

[34] Qoraychy IE, Fekhaoui M, Abidi AE, Yahyaoui A. Biometry and demography of *Procambarus clarkii* in Rharb Region, Morocco. Int. J. Bioflux Soc. 2015; 8:751–760.

[35] Morgan LA, Buttemer WA. Predation by the non-native fish *Gambusia holbrooki* on small *Litoria aurea* and *L*. *dentata* tadpoles. Aust. Zool. 1996; 30(2):143–149.

[36] Cruz MJ, Rebelo R. Vulnerability of Southwest Iberian amphibians to an introduced crayfish, *Procambarus clarkii*. Amphib.-Reptilia. 2005; 26(3):293–303.

[37] Ben Hassine J, Escoriza D. *Bufo spinosus* in Tunisia: new data on occurrence, parasitism and tadpole morphology. Herpetol. Bull. 2014; 127:22–32.

[38] Tingley R, Herman TB. Land-cover data improve bioclimatic models for anurans and turtles at a regional scale. J. Biogeogr. 2009; 36(9):1656–1672.

[39] Hansen MC, Potapov PV, Moore R, Hancher M, Turubanova SA, Tyukavina A, et al High-resolution global maps of 21st-century forest cover change. Science. 2013; 342:850–853. 10.1126/science.1244693 24233722

[40] NASA (National Aeronautics and Space Administration). Advanced Spaceborne Thermal Emission and Reflection Radiometer- Global Digital Elevation Model. Available: http://www.gdem.aster.ersdac.or.jp/search.jsp/. 2016.

[41] Hijmans RJ, Cameron SE, Parra JL, Jones PG, Jarvis A. Very high resolution interpolated global terrestrial climate surfaces. Int. J. Climatol. 2005; 25:1965–1978.

[42] Trabucco A, Zomer RJ. Global Index of aridity (Global Aridity) and Global Potential EvapoTranspiration (Global-PET) geospatial database. Available: http://www.cgiar–csi.org/. 2015.

[43] Quantum-GIS Development Team. QGIS vs 2.18. Open Source Geospatial Foundation Project. Available: http://qgis.osgeo.org. 2016.

[44] Gallien L, Durocher M. Table chronologique du développement chez *Pleurodeles waltlii* (Michah). Bull. Biol. Fr. Belg. 1957; 91:97–114.

[45] Gosner KL. A simplified table for staging anuran embryos and larvae with notes on identification. Herpetologica. 1960; 16:183‒190.

[46] Escoriza D, Ben Hassine J. Phenotypic variability in larvae of two species of Mediterranean spadefoot toad: an approach using linear and geometric morphometrics. Afr. J. Herpetol. 2014; 63(2):152–165.

[47] Iwata H, Ukai Y. SHAPE: a computer program package for quantitative evaluation of biological shapes based on elliptic Fourier descriptors. J. Hered. 2002; 93:384‒385. 12547931 10.1093/jhered/93.5.384

[48] Suzuki R, Shimodaira H. Pvclust: an R package for assessing the uncertainty in hierarchical clustering. Bioinformatics. 2006; 22(12):1540–1542. 10.1093/bioinformatics/btl117 16595560

[49] Anderson MJ. A new method for non-parametric multivariate analysis of variance. Aust. Ecol. 2001; 26:32–46.

[50] Iwata H, Ukai Y. SHAPE. Version 1.3. Available: http://lbm.ab.a.u-tokyo.ac.jp/~iwata/shape/. 2002.

[51] Rasband WS. ImageJ vs 1.50. Available: http://rsb.info.nih.gov/ij/. 2015.

[52] Suzuki R, Shimodaira H. pvclust: Hierarchical Clustering with P-Values via Multiscale Bootstrap Resampling. R package version 2.0.0. Available: http://CRAN.R-project.org/package=pvclust. 2016.

[53] R Core Team. R: A language and environment for statistical computing.: R Foundation for Statistical Computing, Vienna, Austria. Available: http://www.R-project.org. 2016.

[54] McCollum SA, Leimberger JD. Predator-induced morphological changes in an amphibian: predation by dragonflies affects tadpole shape and color. Oecologia. 1997; 109(4):615–621. 28307347 10.1007/s004420050124

[55] Escoriza D. Factors regulating the invasion of two Mediterranean anurans. The role of niche conservatism, species interaction and habitat selection. M. Sc. Thesis, University of Girona. 2015. Available from: http://hdl.handle.net/10803/300902

[56] Fauth JE, Bernardo J, Camara M, Resetarits WJ, van Buskirk J, McCollum SA. Simplifying the jargon of community ecology: a conceptual approach. Am. Nat. 1996; 147:282–286.

[57] Cadotte MW, Dinnage R, Tilman D. Phylogenetic diversity promotes ecosystem stability. Ecology. 2012; 93:223–233.

[58] Hedges SB, Marin J, Suleski M, Paymer M, Kumar S. Tree of life reveals clock-like speciation and diversification. Mol. Biol. Evol. 2015; 32:835–845. 10.1093/molbev/msv037 25739733 PMC4379413

[59] Buisson L, Grenouillet G, Villéger S, Canal J, Laffaille P. Toward a loss of functional diversity in stream fish communities under climate change. Glob. Change Biol. 2013; 19(2):387‒400. 10.1111/gcb.12056 23504778

[60] Schleuter D, Daufresne M, Massol F, Argillier C. A user's guide to functional diversity indices. Ecol. Monogr. 2010; 80(3):469–484.

[61] Dolédec S, Chessel D, Gimaret-Carpentier C. Niche separation in community analysis: a new method. Ecology. 2000; 81(10):2914─2927.

[62] Clarke KR, Gorley RN. PRIMER v6: User Manual/Tutorial. Plymouth: PRIMER-E; 2006.

[63] Legendre P, Galzin R, Harmelin-Vivien ML. Relating behavior to habitat: solutions to the fourth-corner problem. Ecology. 1997; 78(2):547–562.

[64] Lardner B. 2000. Morphological and life history responses to predators in larvae of seven anurans. Oikos. 2000; 88(1):169–180.

[65] Pavoine S, Ollier S, Pontier D, Chessel D. Testing for phylogenetic signal in phenotypic traits: new matrices of phylogenetic proximities. Theor. Popul. Biol. 2008; 73(1):79–91. 10.1016/j.tpb.2007.10.001 18022657

[66] Burnham K, Anderson DR. Model selection and multimodel inference: a practical information-theoretical approach. New York, Springer Verlag; 2002.

[67] Dray S, Dufour AB, Chessel D. The ade4 package-II: Two-table and K-table methods. R News. 2007; 7(2):47–52.

[68] Calenge C. The package adehabitat for the R software: a tool for the analysis of space and habitat use by animals. Ecol. Model. 2006; 197:516–519.

[69] Bartoń K. MuMIn: Multi-Model Inference. R package version 1.15.6. Available: http://CRAN.R-project.org/package=MuMIn. 2016.

[70] Cox N, Chanson J, Stuart S. The status and Distribution of Reptiles and Amphibians of the Mediterranean Basin. Gland and Cambridge: IUCN; 1996.

[71] Gasc JP, Cabela A, Crnobrnja-Isailovic J, Dolmen D, Grossenbacher K, Haffner P, et al Atlas of Amphibians and Reptiles in Europe. Paris: Societas Europaea Herpetologica and Museum National d’Histoire Naturelle; 1997.

[72] Valentine BD, Dennis DM. A comparison of the gill-arch system and fins of three genera of larval salamanders, *Rhyacotriton*, *Gyrinophilus*, and *Ambystoma*. Copeia. 1964; 1964:196–201.

[73] Lanza B, Andreone F, Bologna MA, Corti C, Razzetti E. Fauna d’Italia. Amphibia. Bologna: Calderini; 2007.

[74] Van Buskirk J. Natural variation in morphology of larval amphibians: Phenotypic plasticity in nature?. Ecol. Monogr. 2009; 79(4):681–705.

[75] Escoriza D, Gutiérrez-Rodríguez J, Ben Hassine J, Martínez-Solano I. Genetic assessment of the threatened microendemic *Pleurodeles poireti* (Caudata, Salamandridae), with molecular evidence for hybridization with *Pleurodeles nebulosus*. Conserv. Genet. 2016; 17(6):1445–1458.

[76] Della Bella V, Bazzanti M, Dowgiallo MG, Iberite M. Macrophyte diversity and physico-chemical characteristics of Tyrrhenian coast ponds in central Italy: implications for conservation. Hydrobiologia. 2008; 597(1):85–95.

[77] Caut S, Angulo E, Díaz-Paniagua C, Gómez-Mestre I. Plastic changes in tadpole trophic ecology revealed by stable isotope analysis. Oecologia. 2013; 173(1):95–105. 10.1007/s00442-012-2428-3 22915331

[78] Wilson RS, Franklin CE. Effect of ontogenetic increases in body size on burst swimming performance in tadpoles of the striped marsh frog, *Limnodynastes peronii*. Physiol. Biochem. Zool. 2000; 73(2):142–152. 10.1086/316730 10801392

[79] Skelly DK. Tadpole communities: pond permanence and predation are powerful forces shaping the structure of tadpole communities. Am. Sci. 1997; 85(1):36–45.

[80] Kulkarni SS, Gómez-Mestre I, Moskalik CL, Storz BL, Buchholz DR. Evolutionary reduction of developmental plasticity in desert spadefoot toads. J. Evol. Biol. 2011; 24(11):2445–2455. 10.1111/j.1420-9101.2011.02370.x 21883613

[81] Escoriza D. New data on larval development in *Pelobates varaldii*. Herpetol. Bull. 2013; 125:10–13.

[82] Grillitsch B, Grillitsch H, Splechtna H. The tadpole of *Bufo brongersmai* Hoogmoed 1972. Amphib.-Reptilia. 1989; 10(3):215–229.

[83] Cheddadi R, Lamb HF, Guiot J, Van Der Kaars S. Holocene climatic change in Morocco: a quantitative reconstruction from pollen data. Clim. Dyn. 1998; 14(12):883–890.

[84] Rodríguez-Sánchez F, Arroyo J. Reconstructing the demise of Tethyan plants: climate-driven range dynamics of *Laurus* since the Pliocene. Glob. Ecol. Biogeogr. 2008; 17(6):685–695.

[85] Sandel B, Arge L, Dalsgaard B, Davies RG, Gaston KJ, Sutherland WJ, et al The influence of Late Quaternary climate-change velocity on species endemism. Science. 2011; 334(6056):660–664. 10.1126/science.1210173 21979937

[86] Wassens S, Walcott A, Wilson A, Freire R. Frog breeding in rain-fed wetlands after a period of severe drought: implications for predicting the impacts of climate change. Hydrobiologia. 2013; 708(1):69–80.

[87] Martínez-Solano I, Gonçalves HA, Arntzen JW, García-París M. Phylogenetic relationships and biogeography of midwife toads (Discoglossidae: *Alytes*). J. Biogeogr. 2004; 31(4):603–618.

[88] Escoriza D, Comas MM, Donaire D, Carranza S. (2006). Rediscovery of *Salamandra algira* Bedriaga, 1833 from the Beni Snassen massif (Morocco) and phylogenetic relationships of North African *Salamandra*. Amphib.-Reptilia. 2006; 27:448–455.

[89] Recuero E, Canestrelli D, Vörös J, Szabó K, Poyarkov NA, Arntzen JW, et al Multilocus species tree analyses resolve the radiation of the widespread *Bufo bufo* species group (Anura, Bufonidae). Mol. Phylogenet. Evol. 2012; 62(1):71–86. 10.1016/j.ympev.2011.09.008 21964513

[90] Usseglio-Polatera PH. Theoretical habitat templets, species traits, and species richness: aquatic insects in the Upper Rhône River and its floodplain. Freshw. Biol. 1994; 31(3):417–437.

[91] Rhazi L, Grillas P, Rhazi M, Aznar JC. Ten-year dynamics of vegetation in a Mediterranean temporary pool in western Morocco. Hydrobiologia. 2009; 634(1):185‒194.

[92] Díaz-Paniagua C. Temporal segregation in larval amphibian communities in temporary ponds at a locality in SW Spain. Amphib.-Reptilia. 1988; 9(1):15–26.

[93] Sazima I, Eterovick PC. Structure of an anuran community in a montane meadow in southeastern Brazil: effects of seasonality, habitat, and predation. Amphib.-Reptilia. 2000; 21(4):439–461.

[94] Fox BJ. Niche parameters and species richness. Ecology. 1981; 62:1415–1425.

[95] Babbitt KJ, Baber MJ, Tarr TL. Patterns of larval amphibian distribution along a wetland hydroperiod gradient. Can. J. Zool. 2003; 81(9):1539–1552.

[96] Srivastava DS, Cadotte MW, MacDonald AA, Marushia RG, Mirotchnick N. Phylogenetic diversity and the functioning of ecosystems. Ecol. Lett. 2012; 15(7):637–648. 10.1111/j.1461-0248.2012.01795.x 22583836

[97] Waringer-Löschenkohl A. An experimental study of microhabitat selection and microhabitat shifts in European tadpoles. Amph.-Reptilia. 1988; 9(3):219–236.

[98] Peltzer PM, Lajmanovich RC, Attademo AM, Beltzer AH. Diversity of anurans across agricultural ponds in Argentina. Biodivers. Conserv. 2006; 15(11):3499–3513.

[99] Werner EE, Skelly DK, Relyea RA, Yurewicz KL. Amphibian species richness across environmental gradients. Oikos. 2007; 116(10):1697‒1712.

