# Supplementary material for: Diversity of Guilds of Amphibian Larvae in North-Western Africa
Source: PLoS One. 2017 Jan 26;12(1):e0170763. doi: 10.1371/journal.pone.0170763 (PMC5268446; doi:10.1371/journal.pone.0170763)
Supplement: S1 Table — Table A. Sites and species occurrence. Table B. Larvae traits. Table C. PERMANOVA results assessing intraspecific body shape variation. Table D. Canonical correlation between environmental variables and species occurrence. (DOCX) [file pone.0170763.s001.docx]

Table A. Sites and aquatic habitats. Type, type of aquatic habitat, SPO (stream pool), SPR (spring), TPO (temporary pond), PPO (permanent pond); SAR, surface area (m^2^); DEP, average depth (cm); EVE, emergent vegetation (proportion of surface cover); WTE, water temperature (ºC); ELE, elevation (m above sea level); MAT, mean annual temperature (ºC); AIN, aridity index; FOR, percentage of forest cover (30 m); PRE, predators, 0 (absence), 1 (large native arthropoda: Branchiopoda, Insecta, Malacostraca), 2 (alien fish and crayfish), 3 (natricine snakes and turtles); EFF, sampling effort (number of dip nets); SPE, species co-occurrence. Urodela: PNE, *Pleurodeles nebulosus*; PPO, *Pleurodeles poireti*; PWA, *Pleurodeles waltl*; SAL, *Salamandra algira*; Anura: AMA, *A. mauritanicus*; AMU, *A. maurus*; BBO, *B. boulengeri*; BBR, *B. brongersmai*; BSP, *B. spinosus*; DPI, *D. pictus*; DSC, *D. scovazzi*; HMA, *H. aff. meridionalis* (eastern form); HME, *H. meridionalis*; PSA, *P. saharicus*; PVA, *P. varaldii*. The asterisk indicated populations used for morphological analysis.

| Type | SAR | DEP | EVE | WTE | ELE | MAT | AIN | FOR | PRE | EFF | SPE |
| --- | --- | --- | --- | --- | --- | --- | --- | --- | --- | --- | --- |
| SPR | 0.64 | 41 | 0.5 | 22.0 | 40 | 21.2 | 0.05 | 0 | 0 | 1 | DPI |
| SPR | 0.64 | 47 | 0.0 | 22.4 | 27 | 19.4 | 0.13 | 0 | 0 | 1 | DPI*, PSA |
| SPR | 0.64 | 80 | 0.0 | 21.0 | 18 | 19.4 | 0.13 | 0 | 0 | 1 | DPI, PSA |
| SPR | 0.64 | 105 | 0.0 | 20.8 | 46 | 20.0 | 0.12 | 0 | 0 | 1 | DPI |
| TPO | 0.65 | 7 | 0.0 | 14.7 | 1208 | 13.1 | 0.39 | 78 | 0 | 1 | DPI |
| SPR | 1.00 | 12 | 0.0 | 9.0 | 1952 | 9.5 | 0.45 | 12 | 0 | 1 | AMU*, SAL |
| SPO | 1.26 | 6 | 0.0 | 6.1 | 458 | 16.2 | 0.76 | 74 | 0 | 1 | SAL |
| SPO | 1.35 | 9 | 0.0 | 14.2 | 500 | 15.4 | 0.78 | 75 | 0 | 1 | SAL |
| SPO | 1.40 | 10 | 0.1 | 12.0 | 1222 | 14.5 | 0.34 | 30 | 0 | 1 | PSA*, SAL |
| TPO | 1.42 | 6 | 0.7 | 23.6 | 9 | 18.1 | 0.49 | 3 | 0 | 1 | DPI |
| SPR | 1.52 | 31 | 0.0 | 15.5 | 47 | 18.0 | 0.87 | 0 | 1 | 1 | SAL |
| TPO | 1.56 | 13 | 0.0 | 10.9 | 14 | 17.8 | 0.56 | 0 | 0 | 1 | DSC |
| TPO | 1.71 | 13 | 0.0 | 13.5 | 328 | 16.1 | 0.71 | 89 | 0 | 1 | DSC |
| SPR | 2.24 | 63 | 0.0 | 11.2 | 1024 | 14.4 | 0.89 | 45 | 0 | 2 | SAL |
| SPO | 2.58 | 10 | 0.0 | 18.1 | 535 | 14.5 | 0.81 | 95 | 0 | 2 | PSA |
| SPO | 2.86 | 12 | 0.0 | 11.5 | 535 | 14.5 | 0.81 | 95 | 0 | 2 | SAL |
| SPR | 3.00 | 73 | 0.0 | 9.9 | 1021 | 14.4 | 0.89 | 45 | 0 | 3 | SAL |
| TPO | 3.20 | 22 | 0.1 | 6.7 | 293 | 18.5 | 0.62 | 82 | 0 | 3 | SAL |
| SPR | 3.28 | 112 | 0.0 | 13.3 | 666 | 16.2 | 0.80 | 37 | 1 | 3 | SAL |
| SPR | 3.29 | 12 | 1.0 | 12.8 | 832 | 13.6 | 0.87 | 89 | 0 | 3 | SAL |
| TPO | 3.35 | 6 | 0.1 | 18.7 | 31 | 17.9 | 0.49 | 0 | 1 | 3 | DPI |
| SPO | 3.67 | 27 | 0.0 | 8.8 | 503 | 16.0 | 0.78 | 78 | 0 | 3 | SAL |
| SPO | 3.67 | 15 | 0.0 | 8.2 | 522 | 14.2 | 0.82 | 95 | 0 | 3 | SAL |
| TPO | 3.78 | 11 | 1.0 | 6.5 | 1620 | 11.7 | 0.48 | 0 | 1 | 3 | DSC |
| SPO | 3.92 | 7 | 0.0 | 13.8 | 565 | 16.1 | 0.27 | 60 | 0 | 3 | SAL |
| TPO | 4.00 | 9 | 0.8 | 26.6 | 26 | 18.2 | 0.69 | 0 | 1 | 3 | DPI |
| SPO | 4.03 | 19 | 0.0 | 7.7 | 531 | 16.0 | 0.78 | 95 | 0 | 3 | SAL |
| SPR | 4.42 | 10 | 0.0 | 12.3 | 701 | 14.6 | 0.30 | 0 | 0 | 3 | SAL |
| SPO | 4.01 | 16 | 0.1 | 16.1 | 828 | 13.5 | 0.87 | 78 | 0 | 3 | DPI, PSA* |
| SPO | 4.50 | 8 | 0.0 | 10.3 | 828 | 13.5 | 0.87 | 78 | 0 | 3 | SAL |
| SPR | 4.50 | 87 | 0.2 | 12.9 | 758 | 16.0 | 0.81 | 0 | 1 | 3 | SAL |
| SPO | 5.22 | 9 | 0.6 | 14.0 | 696 | 14.2 | 1.33 | 89 | 0 | 5 | SAL |
| SPO | 5.33 | 7 | 0.0 | 10.3 | 42 | 17.7 | 0.31 | 0 | 1 | 5 | DSC, PSA |
| SPO | 5.33 | 11 | 0.2 | 12.6 | 405 | 16.7 | 0.88 | 44 | 1 | 5 | SAL |
| SPO | 5.76 | 10 | 0.0 | 31.8 | 63 | 19.3 | 0.11 | 0 | 0 | 5 | AMA |
| SPO | 5.89 | 12 | 0.0 | 18.0 | 455 | 16.2 | 0.83 | 52 | 0 | 5 | BSP |
| SPR | 7.80 | 3 | 0.6 | 12.9 | 429 | 15.8 | 0.93 | 0 | 1 | 5 | SAL |
| SPO | 8.50 | 24 | 0.5 | 5.7 | 1620 | 11.7 | 0.48 | 0 | 1 | 5 | PSA |
| SPR | 8.60 | 10 | 0.0 | 30.6 | 25 | 19.7 | 0.13 | 0 | 1 | 5 | DPI |
| SPO | 8.84 | 3 | 0.0 | 27.8 | 68 | 17.8 | 0.57 | 0 | 0 | 5 | AMA |
| TPO | 9.12 | 9 | 0.4 | 14.4 | 655 | 14.8 | 0.82 | 0 | 1 | 5 | DPI |
| TPO | 9.50 | 39 | 0.0 | 17.2 | 31 | 17.9 | 0.49 | 0 | 1 | 5 | DPI |
| SPR | 9.62 | 13 | 0.0 | 11.0 | 1465 | 12.3 | 0.37 | 65 | 1 | 5 | SAL |
| SPR | 10.75 | 8 | 0.5 | 14.0 | 860 | 15.2 | 0.28 | 0 | 1 | 5 | SAL |
| SPR | 11.18 | 70 | 0.3 | 16.0 | 442 | 16.3 | 0.89 | 0 | 1 | 10 | DPI, SAL |
| TPO | 12.00 | 7 | 0.8 | 22.1 | 164 | 17.7 | 0.36 | 0 | 1 | 10 | DPI, PSA |
| TPO | 12.35 | 13 | 0.01 | 14.9 | 621 | 15.3 | 1.01 | 82 | 1 | 10 | DPI |
| SPO | 12.68 | 17 | 1.00 | 18.8 | 8 | 18.0 | 0.37 | 0 | 1 | 10 | HME* |
| SPO | 14.90 | 19 | 0.76 | 19.2 | 12 | 18.0 | 0.36 | 0 | 1 | 10 | HME |
| TPO | 180.00 | 15 | 0.6 | 28.5 | 29 | 17.8 | 0.22 | 0 | 1 | 20 | AMA, DPI |
| TPO | 15.22 | 13 | 0.2 | 17.2 | 467 | 16.2 | 0.83 | 89 | 0 | 10 | DPI, PSA |
| TPO | 15.68 | 12 | 0.7 | 13.1 | 305 | 17.1 | 0.72 | 0 | 1 | 10 | DPI |
| TPO | 16.00 | 26 | 0.0 | 28.8 | 40 | 21.1 | 0.06 | 0 | 0 | 10 | BBO |
| TPO | 3522.50 | 16 | 0.9 | 19.6 | 95 | 17.6 | 0.47 | 0 | 1 | 60 | DPI, PNE |
| TPO | 180.00 | 16 | 0.6 | 26.6 | 231 | 17.1 | 0.30 | 0 | 1 | 20 | DPI, PNE |
| SPO | 17.00 | 11 | 0.3 | 19.0 | 136 | 17.3 | 0.23 | 0 | 1 | 10 | AMA |
| TPO | 17.23 | 33 | 0.2 | 11.9 | 28 | 18.4 | 0.69 | 0 | 1 | 10 | DPI, PPO |
| TPO | 196.00 | 17 | 0.0 | 21.2 | 9 | 18.8 | 0.15 | 0 | 0 | 20 | BBO |
| TPO | 689.49 | 17 | 0.9 | 21.9 | 32 | 17.8 | 0.45 | 0 | 1 | 40 | DPI, PNE |
| TPO | 17.81 | 18 | 0.0 | 18.2 | 31 | 17.9 | 0.49 | 0 | 1 | 10 | DPI |
| TPO | 18.24 | 12 | 0.05 | 20.5 | 856 | 16.1 | 0.37 | 18 | 1 | 10 | DSC |
| TPO | 18.50 | 21 | 0.1 | 21.6 | 112 | 17.8 | 0.65 | 0 | 1,3 | 10 | DPI, HMA |
| SPO | 19.32 | 7 | 0.8 | 15.2 | 300 | 16.6 | 1.02 | 34 | 1,3 | 10 | DPI, SAL |
| SPR | 20.54 | 26 | 1.0 | 11.3 | 1465 | 12.3 | 0.37 | 65 | 1 | 10 | AMU*, SAL |
| TPO | 21.96 | 121 | 0.1 | 18.2 | 197 | 17.8 | 0.64 | 0 | 1 | 10 | DPI, HMA |
| TPO | 22.00 | 19 | 0.7 | 15.7 | 1 | 18.2 | 0.71 | 0 | 1 | 10 | DPI |
| TPO | 2460.00 | 24 | 0.2 | 24.0 | 48 | 17.9 | 0.19 | 0 | 1 | 60 | AMA, HMA, PSA |
| TPO | 26.00 | 12 | 0.9 | 15.2 | 172 | 17.5 | 0.67 | 0 | 1 | 10 | DPI, PNE |
| TPO | 26.21 | 25 | 0.3 | 16.8 | 12 | 18.0 | 0.46 | 0 | 1 | 10 | HMA, PNE |
| TPO | 28.00 | 38 | 0.3 | 15.3 | 1120 | 14.0 | 0.87 | 6 | 1 | 10 | DSC*, SAL |
| TPO | 28.20 | 30 | 0.3 | 11.2 | 7 | 17.8 | 0.56 | 0 | 1 | 10 | DSC* |
| TPO | 28.71 | 18 | 0.9 | 12.2 | 1470 | 12.3 | 0.37 | 65 | 1 | 10 | PSA, SAL |
| TPO | 138.00 | 30 | 0.5 | 23.5 | 16 | 18.0 | 0.21 | 0 | 1 | 20 | DPI, HMA |
| TPO | 32.00 | 65 | 0.5 | 21.1 | 82 | 18.0 | 0.68 | 0 | 1 | 10 | DPI, HMA |
| TPO | 34.27 | 58 | 0.6 | 12.5 | 749 | 15.9 | 0.85 | 56 | 1 | 10 | DSC*, SAL |
| TPO | 35.28 | 10 | 0.3 | 16.4 | 72 | 18.0 | 0.65 | 89 | 1 | 10 | DPI, PNE |
| TPO | 35.88 | 15 | 0.01 | 16.0 | 141 | 17.7 | 0.69 | 64 | 1 | 10 | DPI |
| TPO | 36.00 | 45 | 0.8 | 17.2 | 495 | 16.6 | 0.75 | 97 | 1 | 10 | DPI, PSA |
| TPO | 36.08 | 91 | 0.1 | 11.2 | 495 | 16.6 | 0.75 | 97 | 1 | 10 | SAL |
| SPO | 38.00 | 19 | 0.7 | 18.3 | 43 | 17.7 | 0.23 | 0 | 1 | 10 | DPI, PNE |
| TPO | 40.54 | 8 | 0.5 | 24.8 | 701 | 14.6 | 0.30 | 0 | 1 | 10 | DPI* |
| TPO | 40.98 | 13 | 0.0 | 17.1 | 109 | 17.4 | 0.29 | 0 | 1 | 10 | DPI |
| TPO | 41.80 | 30 | 1.0 | 15.1 | 343 | 17.0 | 0.63 | 0 | 1 | 10 | DPI, HMA |
| TPO | 42.00 | 47 | 0.4 | 26.6 | 20 | 18.0 | 0.37 | 4 | 1 | 10 | HME, PSA |
| TPO | 46.92 | 16.2 | 0.7 | 17.4 | 387 | 16.8 | 0.70 | 74 | 1 | 10 | DPI |
| SPO | 48.30 | 15.2 | 0.0 | 16.2 | 1609 | 10.5 | 0.91 | 71 | 1 | 10 | AMU* |
| TPO | 51.10 | 21 | 0.8 | 12.2 | 433 | 15.8 | 0.71 | 0 | 1 | 10 | DPI |
| TPO | 52.51 | 48 | 0.7 | 15.8 | 197 | 17.6 | 0.75 | 24 | 1 | 10 | HMA, PSA* |
| TPO | 53.50 | 19 | 0.1 | 16.7 | 97 | 17.8 | 0.62 | 0 | 1 | 10 | AMA, DPI |
| TPO | 55.70 | 19 | 0.7 | 20.1 | 19 | 18.0 | 0.52 | 0 | 1 | 10 | DPI, PNE |
| TPO | 56.25 | 33 | 0.8 | 24.3 | 20 | 18.0 | 0.37 | 4 | 1 | 10 | HME |
| TPO | 57.40 | 10 | 0.1 | 26.2 | 29 | 17.9 | 0.49 | 0 | 1 | 10 | DPI, HMA, PNE |
| TPO | 57.40 | 40 | 1.0 | 14.6 | 1173 | 14.0 | 0.87 | 0 | 1 | 10 | AMU, PSA*, SAL |
| TPO | 57.40 | 42 | 0.8 | 24.0 | 420 | 17.8 | 0.12 | 0 | 1 | 10 | AMA, PSA* |
| SPO | 62.00 | 6 | 0.2 | 19.5 | 1322 | 17.0 | 0.20 | 0 | 1 | 10 | AMA* |
| TPO | 62.98 | 15 | 0.7 | 16.3 | 239 | 17.3 | 0.67 | 0 | 1 | 10 | PPO, PSA |
| TPO | 63.00 | 106 | 0.4 | 18.7 | 78 | 18.0 | 0.68 | 0 | 1 | 10 | DPI, HMA |
| TPO | 66.26 | 69 | 0.4 | 17.0 | 121 | 17.8 | 0.62 | 0 | 1 | 10 | DPI, HMA, PNE |
| PPO | 69.00 | 16 | 1.0 | 17.5 | 674 | 15.2 | 0.80 | 90 | 1 | 10 | PSA* |
| TPO | 70.00 | 9 | 0.2 | 14.2 | 100 | 18.2 | 0.46 | 0 | 1 | 10 | HME* |
| TPO | 72.00 | 21 | 0.9 | 15.9 | 441 | 17.6 | 0.20 | 0 | 1 | 10 | AMA, HMA |
| TPO | 72.33 | 38 | 0.6 | 14.9 | 196 | 17.6 | 0.75 | 24 | 1 | 10 | DPI, HMA |
| TPO | 78.75 | 10 | 0.8 | 16.2 | 61 | 17.9 | 0.66 | 0 | 1 | 10 | DPI |
| TPO | 83.52 | 22 | 0.2 | 18.1 | 106 | 17.5 | 0.26 | 0 | 1 | 10 | DPI, PNE |
| TPO | 84.00 | 6 | 0.01 | 23.8 | 199 | 17.7 | 0.42 | 0 | 1 | 10 | BBO, PNE |
| TPO | 90.00 | 11 | 0.1 | 22.6 | 39 | 18.2 | 0.59 | 0 | 1 | 10 | AMA, DPI, HMA |
| TPO | 90.00 | 18 | 0.9 | 19.2 | 26 | 18.2 | 0.69 | 0 | 1 | 10 | DPI, PSA |
| TPO | 90.00 | 25 | 0.1 | 20.1 | 112 | 17.8 | 0.65 | 0 | 1 | 10 | AMA, DPI, HMA |
| TPO | 91.00 | 40 | 0.0 | 20.3 | 7 | 18.0 | 0.36 | 0 | 1 | 10 | AMA, PSA |
| TPO | 94.53 | 30 | 0.9 | 13.0 | 23 | 18.1 | 0.69 | 0 | 1 | 10 | DPI, HMA, PNE |
| TPO | 97.24 | 16 | 0.3 | 16.7 | 1 | 18.2 | 0.71 | 0 | 1 | 10 | PNE |
| TPO | 98.00 | 30 | 0.9 | 16.2 | 10 | 18.0 | 0.48 | 0 | 1 | 10 | DPI, HMA, PNE |
| TPO | 100.00 | 13 | 0.7 | 21.6 | 9 | 17.8 | 0.39 | 0 | 1 | 20 | DPI, PNE |
| TPO | 108.00 | 22 | 0.9 | 18.9 | 25 | 18.0 | 0.50 | 0 | 1 | 20 | DPI, HMA, PNE |
| TPO | 108.00 | 109 | 0.1 | 21.2 | 78 | 18.0 | 0.68 | 0 | 1 | 20 | DPI, HMA |
| TPO | 118.52 | 33 | 0.3 | 16.8 | 24 | 18.4 | 0.60 | 0 | 1 | 20 | DPI, PPO, PSA |
| TPO | 126.00 | 19 | 0.2 | 24.1 | 59 | 17.8 | 0.37 | 0 | 1 | 20 | BBO, DPI* |
| TPO | 127.40 | 11 | 0.9 | 20.5 | 134 | 17.7 | 0.60 | 0 | 1 | 20 | DPI |
| TPO | 129.98 | 17 | 0.6 | 18.6 | 13 | 18.0 | 0.45 | 0 | 1 | 20 | AMA, DPI, PNE |
| TPO | 134.97 | 25 | 0.5 | 9.8 | 1391 | 12.6 | 0.36 | 0 | 1 | 20 | HME, SAL |
| TPO | 135.00 | 17 | 0.6 | 17.6 | 6 | 18.2 | 0.70 | 0 | 1 | 20 | DPI, PNE |
| TPO | 137.83 | 62 | 0.1 | 15.9 | 81 | 17.9 | 0.68 | 0 | 1 | 20 | DPI, HMA |
| TPO | 143.00 | 16 | 0.1 | 21.9 | 595 | 15.8 | 0.93 | 95 | 1 | 20 | DPI, HMA |
| SPR | 144.35 | 11 | 0.1 | 16.0 | 1597 | 11.5 | 0.39 | 83 | 1 | 20 | DSC |
| TPO | 145.20 | 25 | 0.9 | 11.2 | 409 | 16.8 | 0.70 | 74 | 1 | 20 | DPI |
| TPO | 147.15 | 84 | 0.1 | 13.4 | 7 | 18.4 | 0.61 | 0 | 1 | 20 | DPI*, HMA, PSA* |
| TPO | 150.00 | 80 | 0.1 | 13.4 | 197 | 17.6 | 0.75 | 24 | 1 | 20 | PPO, PSA |
| PPO | 160.00 | 46 | 0.3 | 21.2 | 93 | 19.2 | 0.47 | 0 | 1 | 20 | PWA |
| TPO | 168.00 | 10 | 0.8 | 23.5 | 19 | 18.0 | 0.52 | 0 | 1 | 20 | DPI, HMA, PNE |
| SPO | 168.00 | 37 | 0.6 | 18.7 | 63 | 19.3 | 0.11 | 0 | 1 | 20 | PSA |
| TPO | 175.04 | 27 | 0.8 | 14.3 | 14 | 18.1 | 0.62 | 0 | 1 | 20 | HME, PWA |
| TPO | 176.00 | 39 | 0.5 | 10.3 | 336 | 17.8 | 0.69 | 0 | 1 | 20 | DSC, SAL |
| TPO | 178.60 | 6 | 0.2 | 20.2 | 10 | 17.6 | 0.41 | 0 | 1 | 20 | DPI |
| TPO | 200.00 | 22 | 0.6 | 14.4 | 1110 | 11.9 | 0.74 | 85 | 1 | 20 | HMA |
| TPO | 202.50 | 20 | 0.6 | 18.3 | 31 | 17.9 | 0.49 | 0 | 1,2 | 20 | DPI, PSA |
| TPO | 203.00 | 17 | 0.1 | 16.8 | 120 | 17.9 | 0.39 | 0 | 1 | 20 | BBO*, DPI |
| TPO | 204.00 | 17 | 0.7 | 17.4 | 44 | 18.2 | 0.59 | 0 | 1 | 20 | DPI, PNE |
| TPO | 205.30 | 39 | 0.5 | 16.6 | 19 | 18.0 | 0.19 | 0 | 1 | 20 | DPI, HMA, PNE |
| PPO | 212.50 | 103 | 0.5 | 12.4 | 738 | 15.6 | 0.82 | 45 | 1 | 20 | DSC, HME, PSA* |
| TPO | 213.30 | 43 | 1.0 | 10.6 | 1006 | 14.6 | 0.87 | 85 | 1,3 | 20 | HME, PSA, SAL |
| TPO | 216.00 | 18 | 0.2 | 10.0 | 558 | 16.0 | 0.88 | 62 | 1 | 20 | DPI |
| PPO | 219.30 | 69 | 0.5 | 18.1 | 43 | 17.7 | 0.23 | 0 | 1 | 20 | DPI, PNE |
| TPO | 224.00 | 32 | 0.8 | 21.0 | 346 | 16.5 | 0.27 | 0 | 0 | 20 | BBO* |
| TPO | 230.40 | 15 | 0.9 | 16.7 | 2 | 18.1 | 0.59 | 0 | 1 | 20 | DPI, PPO |
| TPO | 230.74 | 17 | 0.1 | 12.8 | 876 | 14.0 | 0.91 | 0 | 1 | 20 | DPI* |
| TPO | 233.26 | 54 | 0.8 | 17.9 | 86 | 17.5 | 0.26 | 0 | 1 | 20 | AMA, DPI, HMA |
| TPO | 234.00 | 16 | 0.7 | 20.4 | 130 | 17.7 | 0.64 | 0 | 1 | 20 | DPI, HMA, PNE |
| TPO | 237.76 | 15 | 0.8 | 17.3 | 0 | 18.1 | 0.62 | 0 | 1 | 20 | DSC*, HME |
| TPO | 239.51 | 21 | 0.2 | 17.6 | 103 | 17.9 | 0.29 | 0 | 1 | 20 | HME |
| TPO | 245.00 | 11 | 0.7 | 21.6 | 36 | 18.2 | 0.60 | 0 | 1 | 20 | DPI, HMA, PNE |
| TPO | 246.00 | 19 | 0.3 | 24.7 | 130 | 17.9 | 0.40 | 0 | 1 | 20 | BBO, PNE |
| SPO | 254.80 | 12 | 0.0 | 24.4 | 72 | 18.8 | 0.13 | 0 | 1 | 20 | AMA*, BBO*, PSA |
| TPO | 255.00 | 20 | 0.3 | 17.6 | 441 | 17.6 | 0.20 | 0 | 1 | 20 | DPI, HMA |
| TPO | 255.00 | 28 | 0.9 | 19.2 | 112 | 17.8 | 0.61 | 0 | 1 | 20 | DPI*, HMA, PNE |
| TPO | 258.52 | 39 | 0.2 | 17.1 | 19 | 18.0 | 0.19 | 0 | 1 | 20 | DPI, HMA |
| TPO | 266.61 | 17 | 0.6 | 23.2 | 26 | 18.2 | 0.69 | 0 | 1 | 20 | BBO*, DPI* |
| TPO | 275.00 | 22 | 0.1 | 19.2 | 118 | 17.4 | 0.28 | 0 | 1 | 20 | DPI, PNE |
| TPO | 280.00 | 41 | 0.4 | 15.2 | 441 | 17.6 | 0.20 | 0 | 1 | 20 | AMA, DPI, HMA |
| TPO | 293.68 | 66 | 0.6 | 13.5 | 119 | 18.3 | 0.65 | 45 | 1 | 20 | PNE, PSA* |
| TPO | 294.00 | 17 | 0.3 | 27.6 | 145 | 18.0 | 0.40 | 0 | 1 | 20 | DPI, PNE |
| TPO | 320.00 | 11 | 0.1 | 26.5 | 207 | 17.7 | 0.45 | 0 | 1 | 20 | PNE |
| TPO | 324.50 | 40 | 0.9 | 14.7 | 18 | 18.0 | 0.69 | 0 | 1 | 20 | PSA |
| TPO | 325.00 | 16 | 0.8 | 16.4 | 51 | 18.0 | 0.58 | 0 | 1 | 20 | DPI, HMA |
| TPO | 334.61 | 25 | 0.5 | 13.3 | 21 | 18.3 | 0.62 | 0 | 1,3 | 20 | DPI |
| TPO | 340.00 | 16 | 1.0 | 17.2 | 98 | 18.1 | 0.46 | 0 | 1 | 20 | DSC |
| SPO | 340.22 | 26 | 0.5 | 15.1 | 455 | 16.2 | 0.83 | 52 | 1 | 20 | BSP* |
| TPO | 356.08 | 20 | 0.6 | 23.2 | 99 | 17.6 | 0.23 | 0 | 1 | 20 | BBO, DPI, HMA*, PNE |
| TPO | 357.12 | 28 | 0.8 | 18.1 | 36 | 18.5 | 0.85 | 0 | 1 | 20 | DPI, HMA |
| PPO | 358.00 | 55 | 0.6 | 13.6 | 36 | 18.5 | 0.85 | 0 | 1 | 20 | PNE |
| TPO | 358.25 | 38 | 0.8 | 21.6 | 109 | 17.9 | 0.29 | 0 | 1 | 20 | HME*, PSA, PWA |
| TPO | 361.00 | 27 | 0.6 | 8.2 | 862 | 14.3 | 0.93 | 78 | 1 | 20 | DPI, SAL |
| TPO | 374.00 | 19 | 0.7 | 20.0 | 34 | 18.9 | 0.31 | 0 | 1 | 20 | BBO, DSC, HME |
| TPO | 376.00 | 32 | 1.0 | 20.4 | 29 | 17.9 | 0.37 | 0 | 1 | 20 | HME |
| TPO | 379.35 | 17 | 0.6 | 14.9 | 14 | 17.9 | 0.61 | 0 | 1 | 20 | HME*, PWA |
| TPO | 380.82 | 46 | 0.7 | 24.0 | 37 | 18.0 | 0.49 | 0 | 1 | 20 | DPI, HMA, PNE |
| TPO | 384.69 | 12 | 0.9 | 11.5 | 0 | 17.8 | 0.56 | 0 | 1 | 20 | DSC, HME |
| TPO | 407.33 | 21 | 0.9 | 11.5 | 0 | 18.0 | 0.63 | 0 | 1 | 20 | DSC*, HME, PWA |
| TPO | 420.86 | 32 | 0.1 | 17.2 | 166 | 17.9 | 0.43 | 0 | 1 | 20 | PVA* |
| TPO | 425.00 | 19 | 0.9 | 15.2 | 637 | 14.7 | 0.82 | 90 | 1 | 20 | DPI, HMA, PNE |
| TPO | 439.79 | 28 | 0.3 | 18.8 | 18 | 17.9 | 0.21 | 0 | 1 | 20 | BBO, DPI |
| TPO | 455.12 | 23 | 0.6 | 16.9 | 383 | 17.2 | 0.33 | 0 | 1 | 20 | HME, PWA |
| TPO | 456.00 | 28 | 0.9 | 21.2 | 162 | 17.7 | 0.36 | 0 | 1 | 20 | AMA, DPI, PSA |
| TPO | 478.83 | 24 | 0.7 | 17.7 | 100 | 18.1 | 0.47 | 0 | 1 | 20 | HME, PVA, PWA |
| TPO | 480.83 | 18 | 0.9 | 21.2 | 272 | 17.4 | 0.34 | 26 | 1 | 20 | HME, PWA |
| TPO | 490.29 | 45 | 0.6 | 13.9 | 257 | 17.5 | 0.68 | 24 | 1 | 20 | DPI*, HMA, PPO, SAL |
| TPO | 496.00 | 25 | 0.2 | 19.7 | 121 | 18.8 | 0.19 | 0 | 0 | 20 | BBO |
| TPO | 496.00 | 46 | 1.0 | 14.4 | 96 | 18.2 | 0.46 | 0 | 1 | 20 | HME, PVA, PWA |
| TPO | 539.55 | 22 | 0.4 | 13.7 | 44 | 18.3 | 0.65 | 0 | 1 | 40 | DPI, PPO |
| TPO | 550.00 | 24 | 1.0 | 15.2 | 14 | 18.0 | 0.87 | 0 | 1 | 40 | DPI |
| TPO | 550.00 | 80 | 0.5 | 17.3 | 112 | 17.8 | 0.61 | 0 | 1 | 40 | DPI, HMA, PNE |
| TPO | 560.30 | 16 | 1.0 | 21.5 | 36 | 18.2 | 0.60 | 0 | 1 | 40 | DPI, PNE |
| TPO | 562.31 | 33 | 0.7 | 13.1 | 35 | 18.2 | 0.65 | 0 | 1,2 | 40 | DPI, HMA |
| TPO | 569.40 | 93 | 0.2 | 13.5 | 168 | 17.1 | 0.71 | 0 | 1 | 40 | DPI, HMA, PPO, PSA* |
| TPO | 572.62 | 11 | 0.9 | 14.1 | 19 | 18.0 | 0.19 | 0 | 1 | 40 | DPI |
| TPO | 573.63 | 18 | 1.0 | 19.1 | 392 | 17.1 | 0.33 | 0 | 1 | 40 | HME, PWA |
| TPO | 586.68 | 21 | 0.1 | 23.6 | 119 | 17.1 | 0.31 | 0 | 1 | 40 | BBO* |
| TPO | 594.48 | 55 | 0.1 | 28.2 | 103 | 18.1 | 0.46 | 0 | 1 | 40 | AMA |
| TPO | 640.00 | 56 | 0.3 | 12.3 | 257 | 17.4 | 0.34 | 0 | 1 | 40 | HME, PWA |
| TPO | 648.40 | 28 | 0.3 | 11.3 | 4 | 17.8 | 0.56 | 0 | 1 | 40 | DSC |
| TPO | 658.00 | 17 | 0.1 | 20.5 | 34 | 18.9 | 0.31 | 0 | 1 | 40 | HME, PWA |
| TPO | 672.00 | 27 | 0.2 | 15.6 | 270 | 17.1 | 0.62 | 0 | 1 | 40 | DPI, HMA |
| TPO | 684.00 | 114 | 0.1 | 17.6 | 111 | 17.8 | 0.63 | 0 | 1,3 | 40 | HMA, PNE |
| TPO | 690.00 | 24 | 0.0 | 7.5 | 1427 | 12.6 | 0.36 | 0 | 1 | 40 | SAL |
| TPO | 705.88 | 13 | 0.5 | 20.5 | 11 | 18.0 | 0.46 | 0 | 1 | 40 | HMA |
| TPO | 707.73 | 26 | 0.8 | 13.5 | 33 | 18.2 | 0.78 | 0 | 1 | 40 | DPI, HMA |
| TPO | 743.45 | 117 | 0.0 | 13.7 | 70 | 17.7 | 0.67 | 0 | 1,3 | 40 | PPO, PSA |
| TPO | 765.00 | 13 | 0.1 | 21.9 | 58 | 17.8 | 0.43 | 0 | 1 | 40 | PNE |
| TPO | 777.30 | 30 | 0.9 | 12.3 | 26 | 18.4 | 0.70 | 0 | 1 | 40 | DPI, PPO |
| TPO | 780.00 | 115 | 0.3 | 18.5 | 112 | 17.8 | 0.61 | 0 | 1 | 40 | DPI, HMA |
| TPO | 786.54 | 30 | 1.0 | 20.7 | 396 | 17.2 | 0.33 | 0 | 1 | 40 | HME, PWA |
| TPO | 800.00 | 29 | 0.6 | 18.0 | 112 | 18.2 | 0.47 | 0 | 1 | 40 | HME, PVA, PWA |
| TPO | 805.00 | 35 | 1.0 | 17.4 | 117 | 18.2 | 0.47 | 0 | 1 | 40 | HME, PVA, PWA |
| TPO | 825.00 | 9 | 0.1 | 17.8 | 204 | 17.8 | 0.42 | 0 | 1 | 40 | BBO, DPI, PNE |
| TPO | 841.64 | 17 | 1.0 | 17.6 | 13 | 18.3 | 0.63 | 0 | 1 | 40 | DPI, PPO |
| SPO | 882.00 | 27 | 0.9 | 19.7 | 15 | 20.2 | 0.07 | 0 | 1 | 40 | BBO |
| TPO | 928.00 | 36 | 0.0 | 15.2 | 200 | 19.5 | 0.08 | 0 | 1 | 40 | BBO |
| TPO | 983.42 | 27 | 0.9 | 15.5 | 62 | 17.9 | 0.60 | 0 | 1 | 40 | HME, PWA |
| TPO | 1000.30 | 27 | 0.9 | 15.2 | 97 | 18.2 | 0.47 | 0 | 1 | 60 | PVA* |
| TPO | 1012.30 | 22 | 1.0 | 16.9 | 0 | 18.3 | 0.47 | 0 | 1,2 | 60 | DSC*, HME* |
| TPO | 1041.60 | 29 | 0.9 | 14.7 | 15 | 18.0 | 0.88 | 3 | 1 | 60 | DPI, PNE |
| TPO | 1081.30 | 41 | 0.4 | 16.8 | 258 | 17.4 | 0.34 | 0 | 1 | 60 | HME, PWA |
| TPO | 1112.32 | 23 | 0.7 | 15.5 | 13 | 17.8 | 0.48 | 0 | 1 | 60 | HME, AMA |
| TPO | 1118.00 | 26 | 1.0 | 20.9 | 100 | 18.1 | 0.47 | 0 | 1 | 60 | HME, PVA, PWA |
| TPO | 1133.90 | 58 | 0.4 | 11.8 | 22 | 18.2 | 0.60 | 0 | 1 | 60 | DSC, HME |
| TPO | 1152.90 | 15 | 0.8 | 15.9 | 11 | 18.4 | 0.72 | 0 | 1 | 60 | DPI, HMA, PPO |
| TPO | 1170.00 | 42 | 1.0 | 18.1 | 102 | 18.1 | 0.47 | 0 | 1 | 60 | HME, PVA, PWA |
| TPO | 1189.37 | 30 | 0.2 | 28.0 | 103 | 18.1 | 0.46 | 0 | 1 | 60 | AMA, PSA*, PWA |
| TPO | 1199.80 | 45 | 0.6 | 10.3 | 18 | 18.4 | 0.62 | 0 | 1 | 60 | DPI, HMA, PPO |
| TPO | 1259.10 | 32 | 1.0 | 17.0 | 21 | 18.5 | 0.65 | 0 | 1 | 60 | DPI*, PPO |
| TPO | 1320.00 | 12 | 1.0 | 7.8 | 978 | 14.6 | 0.88 | 0 | 1 | 60 | DSC, HME, SAL |
| TPO | 1339.50 | 38 | 0.4 | 16.2 | 76 | 18.2 | 0.47 | 0 | 1,3 | 60 | PVA, PWA |
| TPO | 1350.00 | 25 | 0.2 | 15.6 | 244 | 17.4 | 0.57 | 0 | 1,3 | 60 | AMA, DPI |
| TPO | 1380.00 | 30 | 0.2 | 21.7 | 146 | 17.7 | 0.61 | 0 | 1,3 | 60 | DPI, HMA, PNE |
| TPO | 1400.00 | 32 | 0.0 | 20.0 | 71 | 18.8 | 0.31 | 0 | 1 | 60 | PWA |
| TPO | 1400.00 | 50 | 1.0 | 16.0 | 103 | 18.1 | 0.46 | 0 | 1 | 60 | HME, PVA, PWA |
| TPO | 1408.00 | 44 | 0.6 | 12.8 | 47 | 18.3 | 0.70 | 0 | 1 | 60 | DPI, HMA |
| TPO | 1416.50 | 32 | 1.0 | 17.1 | 31 | 17.8 | 0.36 | 0 | 1 | 60 | HME*, PWA |
| TPO | 1540.80 | 44 | 0.9 | 16.0 | 175 | 18.8 | 0.54 | 0 | 1,3 | 60 | HME*, PWA |
| TPO | 1555.00 | 17 | 0.9 | 19.7 | 237 | 17.4 | 0.28 | 0 | 1 | 60 | HME, PWA |
| TPO | 1556.80 | 40 | 0.6 | 16.5 | 279 | 17.3 | 0.28 | 0 | 1 | 60 | AMA, HME*, PSA, PWA |
| TPO | 1575.00 | 33 | 1.0 | 20.3 | 87 | 18.2 | 0.47 | 0 | 1 | 60 | HME, PVA, PWA |
| TPO | 1698.00 | 70 | 0.9 | 13.0 | 21 | 18.3 | 0.62 | 0 | 1 | 60 | HMA, PPO |
| TPO | 1700.00 | 26 | 0.0 | 7.9 | 1427 | 12.6 | 0.36 | 0 | 1 | 60 | SAL |
| TPO | 1704.70 | 23 | 0.3 | 13.2 | 47 | 18.1 | 0.64 | 0 | 1 | 60 | DSC, HME |
| TPO | 1732.90 | 29 | 0.7 | 9.0 | 4 | 18.3 | 0.63 | 0 | 1 | 60 | DPI, PPO |
| PPO | 1734.00 | 36 | 0.4 | 13.5 | 113 | 18.0 | 0.61 | 55 | 1 | 60 | DSC*, HME, SAL |
| TPO | 1820.00 | 21 | 0.7 | 17.3 | 20 | 18.0 | 0.37 | 4 | 1 | 60 | HME |
| TPO | 1826.90 | 41 | 1.0 | 18.7 | 146 | 18.2 | 0.43 | 0 | 1 | 60 | HME, PVA |
| TPO | 1870.00 | 35 | 0.9 | 16.5 | 111 | 18.2 | 0.47 | 0 | 1 | 60 | HME, PVA*, PWA |
| TPO | 1872.00 | 14 | 1.0 | 20.5 | 15 | 18.0 | 0.37 | 0 | 1 | 60 | HME, PWA |
| TPO | 1984.00 | 25 | 0.6 | 16.9 | 12 | 18.1 | 0.60 | 0 | 1 | 60 | HME, PWA |
| TPO | 1984.30 | 20 | 0.9 | 14.7 | 32 | 18.3 | 0.47 | 0 | 1 | 60 | HME, PVA* |
| PPO | 2024.00 | 100 | 0.1 | 8.9 | 1090 | 13.4 | 0.94 | 74 | 1 | 60 | BSP, HME, SAL |
| TPO | 2122.20 | 22 | 0.9 | 14.9 | 4 | 18.0 | 0.50 | 19 | 1 | 60 | HME*, PWA |
| TPO | 2128.00 | 15 | 0.4 | 15.8 | 60 | 18.0 | 0.58 | 0 | 1 | 60 | HMA, PNE, PSA |
| TPO | 2201.40 | 30 | 0.6 | 22.4 | 162 | 18.4 | 0.27 | 0 | 1 | 60 | AMA, HME, PSA,PWA |
| TPO | 2205.00 | 22 | 0.9 | 10.5 | 5 | 18.3 | 0.63 | 0 | 1 | 60 | DPI*, HMA, PPO |
| TPO | 2233.80 | 15 | 0.8 | 14.6 | 75 | 18.0 | 0.54 | 0 | 1 | 60 | DPI, PPO |
| TPO | 2306.10 | 24 | 0.6 | 15.6 | 73 | 18.2 | 0.47 | 0 | 1 | 60 | HME, PVA |
| TPO | 2348.20 | 18 | 0.8 | 15.0 | 14 | 18.1 | 0.62 | 0 | 1 | 60 | PWA |
| TPO | 2373.40 | 16 | 0.6 | 20.3 | 23 | 17.6 | 0.68 | 0 | 1 | 60 | DPI, PPO |
| TPO | 2400.00 | 16 | 1.0 | 17.6 | 25 | 17.9 | 0.37 | 0 | 1 | 60 | HME, PWA |
| TPO | 2405.10 | 21 | 1.0 | 18.1 | 18 | 18.0 | 0.37 | 0 | 1 | 60 | DSC, HME |
| TPO | 2530.40 | 26 | 1.0 | 20.7 | 193 | 17.6 | 0.36 | 0 | 1 | 60 | HME, PVA, PWA |
| TPO | 2585.00 | 39 | 0.6 | 19.4 | 215 | 17.3 | 0.29 | 0 | 1 | 60 | PWA |
| TPO | 2691.00 | 24 | 0.9 | 22.3 | 80 | 18.2 | 0.47 | 0 | 1 | 60 | HME, PVA*, PWA |
| TPO | 2774.60 | 23 | 0.7 | 17.7 | 160 | 18.1 | 0.43 | 0 | 1 | 60 | PVA, PWA |
| TPO | 2890.12 | 46 | 0.5 | 15.9 | 160 | 18.1 | 0.44 | 0 | 1 | 60 | PVA, PWA |
| TPO | 2929.00 | 30 | 0.8 | 16.4 | 130 | 17.7 | 0.64 | 0 | 1 | 60 | DPI, HMA, PNE |
| TPO | 2993.40 | 60 | 0.9 | 12.7 | 313 | 17.7 | 0.64 | 0 | 1 | 60 | HME, PWA, SAL |
| TPO | 3116.00 | 127 | 0.1 | 18.1 | 62 | 17.6 | 0.20 | 0 | 1,3 | 60 | AMA |
| PPO | 3168.00 | 32 | 1.0 | 5.1 | 1193 | 13.3 | 0.88 | 0 | 1,3 | 60 | BSP, HME, SAL |
| TPO | 3199.02 | 54 | 0.3 | 18.6 | 136 | 17.3 | 0.23 | 0 | 1,3 | 60 | AMA |
| TPO | 3312.00 | 37 | 0.7 | 16.4 | 209 | 17.3 | 0.30 | 0 | 1 | 60 | HME, PWA |
| TPO | 3417.00 | 13 | 1.0 | 19.9 | 14 | 17.8 | 0.33 | 0 | 1 | 60 | DPI |
| TPO | 3504.00 | 10 | 0.1 | 18.7 | 172 | 17.9 | 0.48 | 0 | 1 | 60 | BBO, DPI |
| TPO | 3525.00 | 37 | 0.8 | 13.3 | 66 | 18.3 | 0.47 | 0 | 1 | 60 | HME*, PVA |
| TPO | 3610.20 | 35 | 0.0 | 22.1 | 117 | 17.1 | 0.30 | 0 | 1 | 60 | BBO |
| TPO | 3660.00 | 21 | 1.0 | 14.8 | 937 | 13.5 | 0.95 | 4 | 1 | 60 | DPI*, HMA, PNE |
| TPO | 3666.00 | 45 | 1.0 | 13.6 | 103 | 18.1 | 0.46 | 0 | 1 | 60 | HME, PVA, PWA |
| TPO | 3838.90 | 26 | 1.0 | 13.9 | 19 | 18.4 | 0.55 | 0 | 1 | 60 | DPI, HMA, PPO |
| TPO | 3840.50 | 31 | 0.1 | 20.4 | 414 | 17.2 | 0.32 | 0 | 1 | 60 | DSC, HME |
| TPO | 3955.40 | 39 | 0.7 | 12.1 | 21 | 18.3 | 0.62 | 0 | 1 | 60 | DPI*, HMA |
| TPO | 4134.00 | 41 | 0.1 | 14.6 | 100 | 18.2 | 0.46 | 0 | 1 | 60 | HME, PVA, PWA |
| TPO | 4256.00 | 14 | 1.0 | 21.2 | 99 | 17.6 | 0.47 | 0 | 1 | 60 | DPI, HMA, PNE |
| TPO | 4261.00 | 28 | 1.0 | 14.6 | 21 | 18.3 | 0.61 | 0 | 1 | 60 | DPI*, HMA, PPO |
| TPO | 4501.30 | 22 | 0.6 | 11.6 | 16 | 18.2 | 0.62 | 0 | 1 | 60 | DPI, HMA, PPO |
| TPO | 4507.60 | 32 | 0.6 | 11.6 | 16 | 18.2 | 0.62 | 0 | 1 | 60 | BBO*, DSC*, HME, PSA*, PWA |
| SPO | 4650.00 | 19 | 0.2 | 15.5 | 1631 | 11.2 | 0.73 | 0 | 1 | 60 | BSP, DSC, HME, PSA |
| TPO | 4725.00 | 71 | 0.4 | 19.5 | 38 | 18.3 | 0.47 | 0 | 1 | 60 | DSC, HME, PVA |
| TPO | 4840.00 | 26 | 1.0 | 20.9 | 361 | 17.2 | 0.34 | 0 | 1 | 60 | HME, PSA, PWA |
| TPO | 5089.90 | 21 | 0.7 | 14.4 | 548 | 15.7 | 0.60 | 0 | 1 | 60 | AMA, DPI |
| TPO | 5430.70 | 60 | 0.0 | 16.6 | 95 | 19.2 | 0.48 | 0 | 1,3 | 60 | HME, PWA |
| TPO | 5775.90 | 25 | 0.7 | 12.2 | 44 | 18.3 | 0.64 | 0 | 1 | 60 | DPI, HMA*, PPO |
| TPO | 5812.90 | 56 | 0.9 | 13.3 | 46 | 18.0 | 0.56 | 0 | 1 | 60 | PPO |
| TPO | 6165.10 | 32 | 1.0 | 19.0 | 273 | 17.4 | 0.34 | 4 | 1 | 60 | HME, PWA |
| TPO | 7124.90 | 43 | 0.6 | 16.7 | 217 | 17.4 | 0.29 | 0 | 1,3 | 60 | PWA |
| TPO | 7630.30 | 34 | 0.8 | 22.0 | 210 | 17.3 | 0.30 | 0 | 1 | 60 | HME, PSA, PWA |
| TPO | 7749.00 | 25 | 0.9 | 16.9 | 25 | 18.2 | 0.60 | 0 | 1,2 | 60 | AMA, DPI |
| TPO | 8165.00 | 4 | 0.2 | 20.5 | 153 | 18.0 | 0.46 | 0 | 1 | 60 | AMA, BBO, DPI |
| TPO | 9112.80 | 24 | 0.8 | 14.9 | 30 | 18.3 | 0.55 | 0 | 1 | 60 | DPI, PNE |
| TPO | 9240.00 | 9 | 0.2 | 18.9 | 187 | 17.3 | 0.27 | 0 | 0 | 60 | BBR* |
| TPO | 9663.10 | 19 | 1.0 | 14.2 | 6 | 18.5 | 0.63 | 0 | 1 | 60 | DPI, PPO |
| TPO | 9842.00 | 17 | 0.8 | 22.8 | 5 | 18.1 | 0.20 | 0 | 1 | 60 | BBO, AMA, DPI |
| TPO | 10239 | 49 | 0.8 | 19.2 | 395 | 17.7 | 0.37 | 0 | 1 | 60 | BBO, HME*, PWA |
| TPO | 10283 | 33 | 0.9 | 18.8 | 408 | 17.1 | 0.33 | 0 | 1 | 60 | HME, PWA |
| TPO | 10650 | 11 | 1.0 | 21.0 | 27 | 19.7 | 0.42 | 0 | 1 | 60 | HME, PWA |
| TPO | 12345 | 39 | 1.0 | 14.4 | 1 | 18.0 | 0.50 | 0 | 1 | 60 | HME*, PVA, PWA |
| TPO | 14105 | 32 | 1.0 | 16.7 | 205 | 17.5 | 0.35 | 0 | 1 | 60 | HME, PSA, PWA |
| TPO | 15140 | 17 | 1.0 | 16.7 | 140 | 17.6 | 0.36 | 0 | 1 | 60 | HME, PWA |
| TPO | 18656 | 28 | 0.1 | 21.6 | 56 | 18.1 | 0.33 | 0 | 1 | 60 | DPI, PSA |
| TPO | 22774 | 28 | 0.9 | 11.8 | 281 | 17.4 | 0.34 | 4 | 1 | 60 | HME, PWA |
| TPO | 23058 | 74 | 0.3 | 15.9 | 32 | 18.3 | 0.47 | 0 | 1 | 60 | AMA*, BBO*, HME, PVA |
| TPO | 26395 | 23 | 0.6 | 13.4 | 1 | 18.0 | 0.60 | 0 | 1 | 60 | DSC, PWA |
| TPO | 26702 | 30 | 0.6 | 12.0 | 19 | 18.4 | 0.60 | 0 | 1 | 60 | DPI, PPO |
| TPO | 27714 | 23 | 0.9 | 17.0 | 367 | 17.4 | 0.32 | 0 | 1 | 60 | HME*, PWA |
| TPO | 28309 | 29 | 0.8 | 14.2 | 306 | 17.4 | 0.33 | 0 | 1 | 60 | HME, PWA |
| TPO | 29275 | 20 | 1.0 | 14.0 | 277 | 17.4 | 0.34 | 12 | 1 | 60 | HME, PWA |
| TPO | 30956 | 17 | 1.0 | 23.0 | 401 | 17.1 | 0.33 | 0 | 1 | 60 | HME, PWA |
| TPO | 33240 | 43 | 0.6 | 13.3 | 21 | 18.0 | 0.61 | 0 | 1 | 60 | HME, PWA |
| TPO | 40828 | 94 | 0.3 | 18.2 | 38 | 18.3 | 0.47 | 0 | 1 | 60 | AMA, HME, PVA, PSA, PWA |
| TPO | 44232 | 20 | 1.0 | 14.7 | 277 | 17.4 | 0.34 | 12 | 1 | 60 | HME, PWA |
| TPO | 57753 | 29 | 0.3 | 15.5 | 0 | 18.0 | 0.50 | 0 | 1 | 60 | HME |
| TPO | 98622 | 11 | 0.7 | 19.7 | 224 | 17.3 | 0.29 | 0 | 1 | 60 | PWA |
| TPO | 119250 | 18 | 0.3 | 21.5 | 134 | 18.6 | 0.30 | 0 | 1 | 60 | DSC, HME, PWA |
| TPO | 160212 | 30 | 0.2 | 14.3 | 222 | 17.3 | 0.29 | 0 | 1 | 60 | PWA |
| TPO | 261888 | 44 | 0.9 | 12.2 | 0 | 18.0 | 0.63 | 0 | 1 | 60 | HME, PWA |
| TPO | 433566 | 26 | 0.4 | 13.3 | 224 | 17.3 | 0.29 | 0 | 1 | 60 | PWA |
| TPO | 748440 | 21 | 0.3 | 17.8 | 219 | 17.3 | 0.29 | 0 | 1 | 60 | PWA |
| PPO | 1089582 | 100 | 0.1 | 14.6 | 1479 | 12.5 | 0.51 | 0 | 1,2,3 | 60 | BBO*, HME, PSA* |

Table B. Species sites (*N*), number of specimens examined by species (*n*) and larval traits.

|  | *N* | *n* | Size (mm^2^) | PC1 shapes | Group | Development (days) | Eye position | Oral apparatus | Color pattern |
| --- | --- | --- | --- | --- | --- | --- | --- | --- | --- |
| *A. mauritanicus* | 31 | 27 | 124.01 | -0.052 | Benthic | 42 | Dorsal | Ventral | Mottled |
| *A. maurus* | 4 | 11 | 352.89 | -0.015 | Benthic | 70 | Dorsal | Ventral | Mottled |
| *B. boulengeri* | 25 | 34 | 186.11 | -0.031 | Benthic | 45 | Dorsal | Ventral | Uniform |
| *B. brongersmai* | 1 | 5 | 25.05 | -0.070 | Benthic | 20 | Dorsal | Ventral | Uniform |
| *B. spinosus* | 5 | 14 | 107.49 | -0.031 | Benthic | 87 | Dorsal | Ventral | Uniform |
| *D. pictus* | 136 | 47 | 172.93 | 0.0160 | Benthic | 60 | Dorsal | Ventral | Bicolored |
| *D. scovazzi* | 29 | 27 | 177.56 | -0.010 | Benthic | 60 | Dorsal | Ventral | Bicolored |
| *H. meridionalis* | 90 | 53 | 261.90 | 0.041 | Nektonic | 90 | Lateral | Terminal | Mottled |
| *H. aff. meridionalis* | 61 | 14 | 274.52 | 0.030 | Nektonic | 90 | Lateral | Terminal | Mottled |
| *P. nebulosus* | 47 | 16 | 482.22 | -0.018 | Pond | 90 | Dorsal | Terminal | Mottled |
| *P. poireti* | 26 | 10 | 454.55 | -0.021 | Pond | 90 | Dorsal | Terminal | Mottled |
| *P. varaldii* | 26 | 57 | 1552.81 | 0.103 | Nektonic | 120 | Dorsal | Terminal | Mottled |
| *P. saharicus* | 46 | 43 | 818.13 | -0.023 | Benthic | 120 | Dorsal | Terminal | Mottled |
| *P. waltl* | 71 | 60 | 828.78 | -0.013 | Pond | 120 | Dorsal | Terminal | Mottled |
| *S. algira* | 43 | 20 | 282.91 | 0.051 | Stream | 120 | Dorsal | Terminal | Uniform |

Table C. PERMANOVA results assessing intraspecific variation based on Fourier coefficients.

|  | Pseudo*-F* | *P* |
| --- | --- | --- |
| *A. maurus* | 0.0008 | 1.00 |
| *A. mauritanicus* | 0.001 | 1.00 |
| *B. boulengeri* | 0.001 | 1.00 |
| *B. brongersmai* | 0.0005 | 1.00 |
| *B. spinosus* | 0.0007 | 1.00 |
| *D. pictus* | 0.003 | 1.00 |
| *D. scovazzi* | 0.002 | 1.00 |
| *H. meridionalis* (western form) | 0.004 | 1.00 |
| *H. aff. meridionalis* (eastern form) | 0.002 | 1.00 |
| *P. nebulosus* | 0.002 | 1.00 |
| *P. poireti* | 0.004 | 1.00 |
| *P. varaldii* | 0.002 | 1.00 |
| *P. saharicus* | 0.002 | 1.00 |
| *P. waltl* | 0.002 | 1.00 |
| *S. algira* | 0.002 | 1.00 |

Table D. Canonical correlation between environmental variables (columns) and species occurrence (rows). Abbreviations are given in S1.

|  | WBS | WTE | EVE | ELE | MAT | AIN | FOR | PPO | SPO | SPR | TPO |
| --- | --- | --- | --- | --- | --- | --- | --- | --- | --- | --- | --- |
| AMA | 0.31 | 0.83 | -0.37 | -0.23 | 0.32 | -0.72 | -0.42 | -0.09 | 0.22 | -0.21 | -0.04 |
| AMU | -0.18 | -0.90 | -0.06 | 3.43 | -3.36 | 0.61 | 0.98 | -0.09 | 0.52 | 2.27 | -1.69 |
| BBO | 0.26 | 0.80 | -0.58 | -0.25 | 0.43 | -0.95 | -0.42 | 0.33 | -0.06 | -0.21 | 0.08 |
| BBR | -0.17 | 0.45 | -0.89 | -0.20 | 0.01 | -1.16 | -0.42 | -0.09 | -0.33 | -0.21 | 0.42 |
| BSP | -0.08 | -0.96 | -0.45 | 1.87 | -1.90 | 1.51 | 0.93 | -0.09 | 1.70 | -0.21 | -1.27 |
| DPI | -0.14 | 0.18 | 0.04 | -0.29 | 0.21 | 0.19 | -0.11 | -0.09 | -0.20 | -0.14 | 0.27 |
| DSC | -0.09 | -0.59 | -0.08 | 0.31 | -0.28 | 0.18 | 0.04 | 0.27 | -0.09 | -0.21 | 0.13 |
| HMA | 0.12 | 0.09 | 0.10 | -0.31 | 0.23 | 0.28 | -0.21 | -0.09 | -0.33 | -0.21 | 0.42 |
| HME | 0.08 | -0.04 | 0.58 | -0.10 | 0.15 | -0.29 | -0.28 | 0.14 | -0.22 | -0.21 | 0.26 |
| PNE | -0.15 | 0.52 | 0.14 | -0.41 | 0.26 | 0.05 | -0.24 | -0.09 | -0.26 | -0.21 | 0.36 |
| PPO | -0.08 | -0.65 | 0.42 | -0.55 | 0.50 | 0.57 | -0.35 | -0.09 | -0.33 | -0.21 | 0.42 |
| PSA | 0.42 | 0.13 | -0.14 | 0.29 | -0.23 | -0.08 | 0.21 | 0.59 | 0.41 | -0.21 | -0.38 |
| PVA | -0.06 | 0.04 | 0.60 | -0.44 | 0.51 | -0.27 | -0.42 | -0.09 | -0.33 | -0.21 | 0.42 |
| PWA | 0.15 | 0.11 | 0.58 | -0.28 | 0.35 | -0.49 | -0.38 | -0.09 | -0.33 | -0.21 | 0.42 |
| SAL | -0.13 | -1.26 | -0.54 | 1.50 | -1.45 | 0.93 | 1.24 | -0.09 | 0.66 | 1.36 | -1.30 |
